# Deep Learning for the Assessment of Alveolar Bone Loss on Intraoral Radiographs: A Systematic Review

**DOI:** 10.3390/diagnostics16162575

**Published:** 2026-08-14

**Authors:** Nada Tawfig Hashim, Bakri Gobara Gismalla, Muhammed Mustahsen Rahman, Riham Mohammed, Vivek Padmanabhan, Md Sofiqul Islam, Rasha Babiker, Mariam Elsheikh, Ayman Ahmed, Bhavna Jha Kukreja

**Affiliations:** 1 Department of Periodontics, RAK College of Dental Sciences, RAK Medical & Health Sciences University, Ras-AL Khaimah 12973, United Arab Emirates; 2 Department of Oral Rehabilitation, Faculty of Dentistry, University of Khartoum, Khartoum 11115, Sudan; 3 Department of Oral Surgery, RAK College of Dental Sciences, RAK Medical & Health Sciences University, Ras-Al Khaimah 12973, United Arab Emirates; 4 Department of Pediatric and Preventive Dentistry, RAK College of Dental Sciences, RAK Medical & Health Sciences University, Ras-Al Khaimah 12973, United Arab Emirates; 5 Department of Operative Dentistry, RAK College of Dental Sciences, RAK Medical and Health Sciences University, Ras-Al Khaimah 12973, United Arab Emirates; sofiqul.islam@rakmhsu.ac.ae; 6 Department of Physiology, RAK College of Medical Sciences, RAK Medical and Health Sciences University, Ras Al Khaimah 11172, United Arab Emirates; 7 Department of Oral &Maxillofacial Surgery, Faculty of Dentistry, University of Khartoum, Khartoum 102, Sudan; 8 Department of Periodontology and Implantology, College of Dentistry, The National Ribat University, Khartoum 11115, Sudan; 9 Department of Preventive Dental Sciences, College of Dentistry, Gulf Medical University, Ajman 4184, United Arab Emirates

**Keywords:** artificial intelligence, deep learning, convolutional neural network, alveolar bone loss, periodontitis, periapical radiography, bitewing radiography, diagnostic imaging, systematic review

## Abstract

**Background**. Radiographic bone loss is a primary determinant of periodontitis stage. Intraoral radiographs (periapical and bitewing) are the standard projections for assessing interproximal bone levels, yet their interpretation is subjective and poorly reproducible, and previous syntheses have pooled intraoral with panoramic imaging. **Objectives**. To appraise and synthesise studies developing or validating deep learning (DL) models for detecting, quantifying, staging or classifying alveolar bone loss on intraoral radiographs. **Methods**. Seven databases and six supplementary sources were searched from 1 January 2015 to 12 June 2026 (PROSPERO CRD420261455818, registered retrospectively). Two reviewers screened, extracted and appraised in duplicate using QUADAS-2 with AI-specific signalling questions, CLAIM and APPRAISE-AI; certainty was rated by GRADE. Heterogeneity precluded pooling; synthesis was narrative. **Results**. Sixteen publications reporting 15 independent datasets (2018–2026) were included (11 periapical, two bitewing, three mixed; 39–21,819 radiographs). The architectures employed comprised classification, segmentation, object-detection, keypoint-localisation and transformer-based models. Accuracy for binary detection ranged from 0.73 to 0.97, Dice coefficients reached ≥0.91, and intraclass correlation with expert measurement was 0.75–0.85. Performance fell for multiclass staging, posterior sites and furcations. Only two studies used an external test set; none was prospective; risk of bias was mostly high or unclear. **Conclusions**. Performance lies within the range observed for calibrated readers, but the evidence is dominated by small, single-centre, retrospective datasets with annotation-based reference standards and almost no external validation; certainty is very low. Deep learning is best regarded as a clinician-supervised adjunct for screening, triage and quality assurance rather than an autonomous diagnostic device.

## 1. Introduction

Periodontitis is a chronic, host-mediated inflammatory disease characterised by progressive destruction of the tooth-supporting apparatus, and severe periodontitis remains among the most prevalent chronic conditions worldwide [1,2]. The 2018 World Workshop redefined periodontitis as a single disease entity staged by severity and complexity and graded by risk of progression [3,4]. Within that framework, interproximal radiographic bone loss (RBL) at the worst-affected site, as a percentage of root length, or in millimetres from the cemento-enamel junction (CEJ) to the alveolar crest, is the primary determinant of stage when clinical attachment level (CAL) data are incomplete, and the ratio of bone loss to patient age is the primary criterion for grade [3]. Radiographic assessment is therefore embedded in the case definition itself rather than being an ancillary investigation.

Intraoral radiography (periapical and bitewing projections) is the standard radiographic method for assessing interproximal bone levels, although periodontal diagnosis and staging require clinical attachment level and a full periodontal examination rather than radiographic assessment alone. Compared with panoramic radiography, intraoral images offer superior spatial resolution, markedly less geometric distortion and better depiction of the CEJ, the crestal cortical plate and furcation entrances [5,6,7]. Periapical radiographs taken with a paralleling technique allow the whole root length to be visualised and the percentage of bone loss to be computed, while vertical bitewings give the most reliable depiction of crestal bone height in moderate disease [6,7]. The trade-off is that a full-mouth series generates fourteen to twenty images per patient, each requiring inspection tooth by tooth and surface by surface.

This is where human interpretation performs least well. Agreement between clinicians on the presence, extent and pattern of bone loss is only moderate, and deteriorates with fatigue, with limited experience, and in anatomically complex regions such as furcations and areas obscured by restorations [8,9,10]. Clinicians also systematically underestimate bone loss on two-dimensional images relative to cone-beam computed tomography, and no routine mechanism exists for auditing the consistency of radiographic diagnosis within or between practices [11]. The consequence is variable staging, variable treatment planning and variable epidemiological estimates.

Deep learning (DL), in which multilayered neural networks learn hierarchical feature representations directly from image data, has become the dominant approach to medical image analysis over the past decade [12,13]. What began as isolated demonstrations of expert-level classification in single specialties has broadened into a general-purpose imaging methodology. Convolutional and, more recently, transformer architectures are now applied across radiology, pathology and ophthalmology to two- and three-dimensional image interpretation, to anatomical and pathological segmentation and mapping, to quantitative measurement of structures that were previously assessed by eye, and to automated report generation, with a growing number of systems cleared by regulators for clinical use [13,14]. Dentistry has followed the same trajectory over the same period, with validated applications in caries detection, tooth numbering and charting, apical lesion identification, root fracture detection, landmark localisation and three-dimensional image segmentation [15,16]. Applied to periodontal radiology, it offers three propositions: reproducibility, because a fixed model returns the same output for the same image; speed, because a full-mouth series can be processed in seconds; and scalability, because retrospective archives can be re-analysed for audit and epidemiology at negligible marginal cost [17].

Most published DL work on periodontal bone loss has used panoramic radiographs [18,19], and existing syntheses have tended to pool panoramic and intraoral studies together [20,21]. This is methodologically problematic. The modalities differ in resolution, in geometric distortion, in visibility of the CEJ and in feasible annotation strategies, so a model validated on panoramic images cannot be assumed to transfer to intraoral images and pooled estimates across modalities are difficult to interpret. The workflows also differ: panoramic imaging is typically a screening projection, whereas intraoral imaging is the projection on which definitive staging decisions are made. A synthesis restricted to intraoral radiography is therefore needed if the evidence is to inform practice.

This systematic review addresses that gap. It identifies, appraises and synthesises the primary studies that developed or validated DL models for assessing alveolar bone loss on intraoral radiographs, characterises the architectures and datasets used, summarises reported diagnostic performance across detection, quantification, staging and pattern classification tasks, evaluates risk of bias and reporting quality, and appraises how close this technology is to clinically deployable practice. Four objectives are addressed in sequence: (i) to characterise the datasets, architectures and reference standards used; (ii) to summarise reported performance separately for each diagnostic task rather than as a single aggregate; (iii) to appraise risk of bias, reporting completeness and methodological quality using instruments designed for artificial intelligence studies; and (iv) to rate the certainty of the evidence for each outcome and judge readiness for clinical use.

## 2. Materials and Methods

### 2.1. Protocol, Registration and Reporting Guidance

This review was designed and conducted in accordance with the Preferred Reporting Items for Systematic Reviews and Meta-Analyses (PRISMA) 2020 statement [22] and the PRISMA extension for diagnostic test accuracy studies (PRISMA-DTA) [23]. The protocol was registered in the International Prospective Register of Systematic Reviews (PROSPERO; registration number CRD420261455818) on 20 July 2026. Registration was retrospective: the record was submitted after the searches, screening, data extraction and risk-of-bias assessment had been completed, and a full chronology of the review, together with a table of all deviations from the registered protocol, is provided as Supplementary File S6. The record was amended on 30 July 2026 (version 1.1) so that the stage-of-review declaration and review start date accurately reflect the conduct of the review; both versions remain publicly visible in the PROSPERO version history. Any deviations from the registered protocol are declared in Section 2.11. The completed PRISMA 2020 checklist is provided as Supplementary File S1 and the full electronic search strategies for every database as Supplementary File S2.

A search of PROSPERO at the time of registration identified three registered reviews addressing artificial intelligence for the radiographic detection of alveolar bone loss. All three are unrestricted as to imaging modality or combine intraoral and extraoral radiographs within a single synthesis. The present review differs in three respects. It is restricted to intraoral (periapical and bitewing) radiography, on the grounds set out in Section 4.2 that panoramic and intraoral images differ materially in spatial resolution, geometric distortion and visibility of the cemento-enamel junction, such that accuracy estimates pooled across modalities are difficult to interpret clinically. It stratifies synthesis by diagnostic task rather than reporting a single aggregate estimate. And it rates certainty of evidence separately for each outcome using GRADE, supported by three complementary appraisal instruments. This overlap is declared on the public PROSPERO record.

### 2.2. Research Question and PICO Framework

The review was structured around a diagnostic accuracy variant of the PICO framework (PIRD: Population, Index test, Reference standard, Diagnosis of interest), presented here in the conventional PICO format for readability. The framework, together with the search concepts derived from each element, is set out in Table 1.

Primary research question: “In patients whose alveolar bone level is assessed on intraoral (periapical and/or bitewing) radiographs, what is the diagnostic performance of deep learning–based image analysis, compared with assessment by calibrated dental clinicians or a clinically derived reference standard, for the detection, quantification, staging and pattern classification of alveolar bone loss?”

Secondary review questions

- Which deep learning architectures and training strategies have been applied to intraoral radiographs for bone loss assessment, and how has this evolved over time?
- How large, how diverse and how well characterised are the datasets used, and were they split at patient level or at image level?
- What reference standards were used to define ground truth, how were annotators calibrated, and how was inter-annotator agreement handled?
- Does model performance differ by radiographic projection (periapical versus bitewing), by tooth type/region, and by task complexity (binary detection versus multiclass staging versus continuous measurement)?
- How do deep learning models compare directly with human readers when both are evaluated on the same test set?
- What is the risk of bias, the completeness of reporting and the overall certainty of the evidence, and what is required before clinical deployment?

### 2.3. Eligibility Criteria

#### 2.3.1. Inclusion Criteria

- Original peer-reviewed research articles, or full conference papers with complete methodological reporting, published in English.
- Use of intraoral radiographs (periapical, bitewing, or both) as the input imaging modality. Studies using mixed datasets were eligible only if intraoral-specific results could be extracted or if intraoral images constituted the substantive majority of the dataset.
- Application of a deep learning architecture (≥2 hidden layers with representation learning from image data). Classical machine learning applied to handcrafted or clinician-measured features was eligible only where it was directly compared with, or embedded within, a deep learning pipeline.
- Alveolar bone loss, radiographic bone level, periodontal bone loss pattern, or radiographically defined periodontitis stage as the target of the model. Studies whose principal target was another condition (for example caries detection, tooth numbering, implant identification or apical lesion detection) were eligible only where a bone loss output was reported separately.
- Reporting of at least one quantitative performance metric evaluated against a defined reference standard.
- Publication between 1 January 2015 and 12 June 2026, reflecting the period in which deep learning became technically feasible for dental radiographic analysis.

Exclusion criteria (the imaging-modality and target-condition criteria are not restated here, because a record failing either was excluded under the corresponding inclusion criterion above; only additional grounds for exclusion are listed)

- Studies using only classical machine learning or image-processing techniques (thresholding, fractal analysis, edge detection, support vector machines on handcrafted features) with no deep learning component.
- Narrative reviews, systematic reviews, editorials, letters, commentaries, conference abstracts without full methods, case reports and technical notes lacking quantitative validation.
- Purely in vitro or phantom studies, and animal studies.
- Studies with no extractable performance metric or no defined reference standard.
- Duplicate publications and companion papers reporting the same dataset and model; in such cases the most complete report was retained.

#### 2.3.2. Index Test (Intervention) Criteria

Eligible index tests are deep learning architectures applied directly to the intraoral radiographic image, operationally defined as models with two or more hidden layers that learn feature representations from image data. Four architecture families are eligible: classification convolutional neural networks (VGG, ResNet, Inception, DenseNet, EfficientNet and comparable networks); encoder–decoder segmentation networks (U-Net, Mask R-CNN, DeepLab, TransUNet); object detectors and keypoint localisers (Faster R-CNN, the YOLO family, Keypoint R-CNN); and vision transformer architectures (ViT, BEiT, DeiT). Hybrid and ensemble pipelines combining these are eligible. Models are eligible irrespective of pretraining strategy, software framework, or whether they were purpose-built for research or commercially developed, including regulator-cleared proprietary systems evaluated as a standalone index test.

Hybrid pipelines in which a deep network performs segmentation or landmark localisation and a conventional algorithm performs the subsequent measurement or classification are eligible, because representation learning from image data occurs within the pipeline. Conversely, studies applying support vector machines, random forests, logistic regression or shallow neural networks to clinician-measured numerical features rather than to image data are not eligible, nor are threshold-based, edge-detection or fractal-dimension analyses. Large language models and generative systems evaluated for text-based diagnostic reasoning rather than radiographic image analysis are excluded.

#### 2.3.3. Reference Standard (Comparator) Criteria

The comparator is the reference standard against which the model was evaluated. Four categories are eligible: assessment of the same intraoral radiograph by one or more calibrated dentists, periodontists or oral and maxillofacial radiologists, whether by single reader, majority vote, consensus discussion or senior adjudication; manual measurement of the distance from the cemento-enamel junction to the alveolar bone crest, or of percentage bone loss relative to root length; a clinical periodontal diagnosis derived from clinical attachment level, probing pocket depth and periodontitis-attributable tooth loss, assigned under the 2018 World Workshop classification or an earlier recognised classification; and, where used, cone-beam computed tomography, surgical exposure or histological confirmation.

Studies are excluded where no reference standard is defined; where the reference standard is described only as unspecified expert opinion with no statement of annotator number, discipline or calibration; where model output is compared solely against another algorithm with no human or clinically derived ground truth at any point; where the reference standard derives from a different imaging modality in a manner that prevents site-level correspondence; where only internal model metrics such as training loss or per-epoch validation accuracy are reported; or where the reference standard was generated wholly or partly by the model under evaluation or an earlier version of it, which constitutes circular validation. Self-reported outcomes, questionnaire-based screening instruments and salivary or serum biomarkers are not accepted as reference standards for radiographic bone level.

#### 2.3.4. Study Design Criteria

Eligible designs are retrospective and prospective diagnostic accuracy studies; cross-sectional and cohort accuracy studies; model development studies incorporating internal validation by hold-out partition, cross-validation or repeated resampling; external validation studies using data from a separate institution, sensor or population; comparative reader studies in which model output and independent human assessment are evaluated on the same test set; and within-study comparisons of competing architectures on a common dataset. Randomised designs, including randomised reader studies and randomised evaluations of artificial-intelligence-assisted versus unassisted interpretation, were eligible and were actively sought; none was identified, and this is itself reported as a finding in Section 4.5.

Excluded designs are narrative reviews, systematic reviews, scoping reviews, meta-analyses, editorials, letters, commentaries, case reports and case series; conference abstracts, posters and technical notes lacking full methods or quantitative validation; purely in vitro, phantom, cadaveric and animal studies; and simulation studies estimating performance on synthetically generated radiographs without evaluation on clinical images. Reviews retrieved by the search were retained for citation chasing but not included as primary studies. Preprints were eligible only where no subsequent peer-reviewed version of the same work existed.

### 2.4. Information Sources and Search Strategy

A comprehensive search was executed by an information specialist in collaboration with two reviewers. The following electronic databases were searched from 1 January 2015 to 12 June 2026: MEDLINE via PubMed, Scopus, Web of Science Core Collection, Embase, IEEE Xplore, ACM Digital Library, and the Cochrane Central Register of Controlled Trials. Because dental artificial intelligence research is frequently disseminated outside the conventional dental literature, the search was supplemented by (i) the first 200 hits of a Google Scholar search sorted by relevance; (ii) the preprint servers arXiv and medRxiv; (iii) the ClinicalTrials.gov and ISRCTN registries for ongoing or unpublished studies; (iv) backward citation chasing of all included articles and of relevant reviews; (v) forward citation chasing using Scopus and Google Scholar “cited by” functions; and (vi) hand-searching of the last five volumes of Journal of Clinical Periodontology, Journal of Periodontology, Journal of Dentistry, Dentomaxillofacial Radiology, Oral Radiology, Journal of Dental Sciences, BMC Oral Health and Diagnostics. Grey literature was searched via OpenGrey and ProQuest Dissertations & Theses. Because full conference papers were eligible, conference proceedings were searched explicitly rather than incidentally: IEEE Xplore and the ACM Digital Library index the computer-science and medical-imaging proceedings in which dental artificial intelligence work most often appears first, and the Conference Proceedings Citation Index within Web of Science and the conference-paper document type within Scopus were searched using the same strategy and the same final search date. Proceedings not indexed on these platforms, including national and regional dental and dental-informatics meetings, were sought only through Google Scholar, grey-literature searching and citation chasing; this residual limitation is acknowledged in Section 4.7. In the event, no full conference paper met the inclusion criteria, and all included reports are peer-reviewed journal articles. Reference management and de-duplication were performed in EndNote 21, with screening conducted in Rayyan.

The strategy was piloted against a set of known eligible studies to confirm that it retrieved each of them before it was executed, and was then adapted to the syntax of each interface. MEDLINE was searched through the PubMed interface as a single search rather than as two independent database searches. Grey and unpublished sources were included deliberately rather than as a formality: models that underperform are rarely submitted or accepted for publication, the published record on this topic contains almost no negative results, and preprint and registry searching is the only partial mitigation available given that funnel plot asymmetry testing is inapplicable in the absence of pooling. The search strategy combined three concept blocks with the Boolean operator AND, with terms within each block combined using OR, and with controlled vocabulary (MeSH/Emtree) exploded and combined with free-text terms searched in title, abstract and keyword fields. Thirteen sources were searched in total: seven electronic databases and six supplementary sources. The PubMed strategy is reproduced in Box 1; the complete strategy for each of the remaining sources is provided in Supplementary File S2.

Box 1 **PubMed search strategy.**

(((“deep learning”[MeSH Terms] OR “deep learning”[tiab] OR “machine learning”[MeSH Terms] OR “artificial intelligence”[MeSH Terms] OR “artificial intelligence”[tiab] OR “neural networks, computer”[MeSH Terms] OR “convolutional neural network*”[tiab] OR CNN[tiab] OR “U-Net”[tiab] OR “Mask R-CNN”[tiab] OR “YOLO”[tiab] OR “vision transformer*”[tiab] OR “computer-aided detection”[tiab] OR “computer-aided diagnos*”[tiab] OR “automated detection”[tiab] OR “semantic segmentation”[tiab]) AND (“alveolar bone loss”[MeSH Terms] OR “alveolar bone loss”[tiab] OR “periodontal bone loss”[tiab] OR “radiographic bone loss”[tiab] OR “bone level”[tiab] OR “crestal bone”[tiab] OR “periodontitis”[MeSH Terms] OR “periodontitis”[tiab] OR “periodontal disease*”[tiab]) AND (“radiography, dental”[MeSH Terms] OR “radiography, bitewing”[MeSH Terms] OR “periapical radiograph*”[tiab] OR “intraoral radiograph*”[tiab] OR “intra-oral radiograph*”[tiab] OR “bitewing*”[tiab] OR “bite-wing*”[tiab] OR “dental radiograph*”[tiab] OR “dental X-ray*”[tiab]))) NOT (“review”[Publication Type] OR “case reports”[Publication Type])

### 2.5. Study Selection

**Figure 1:**
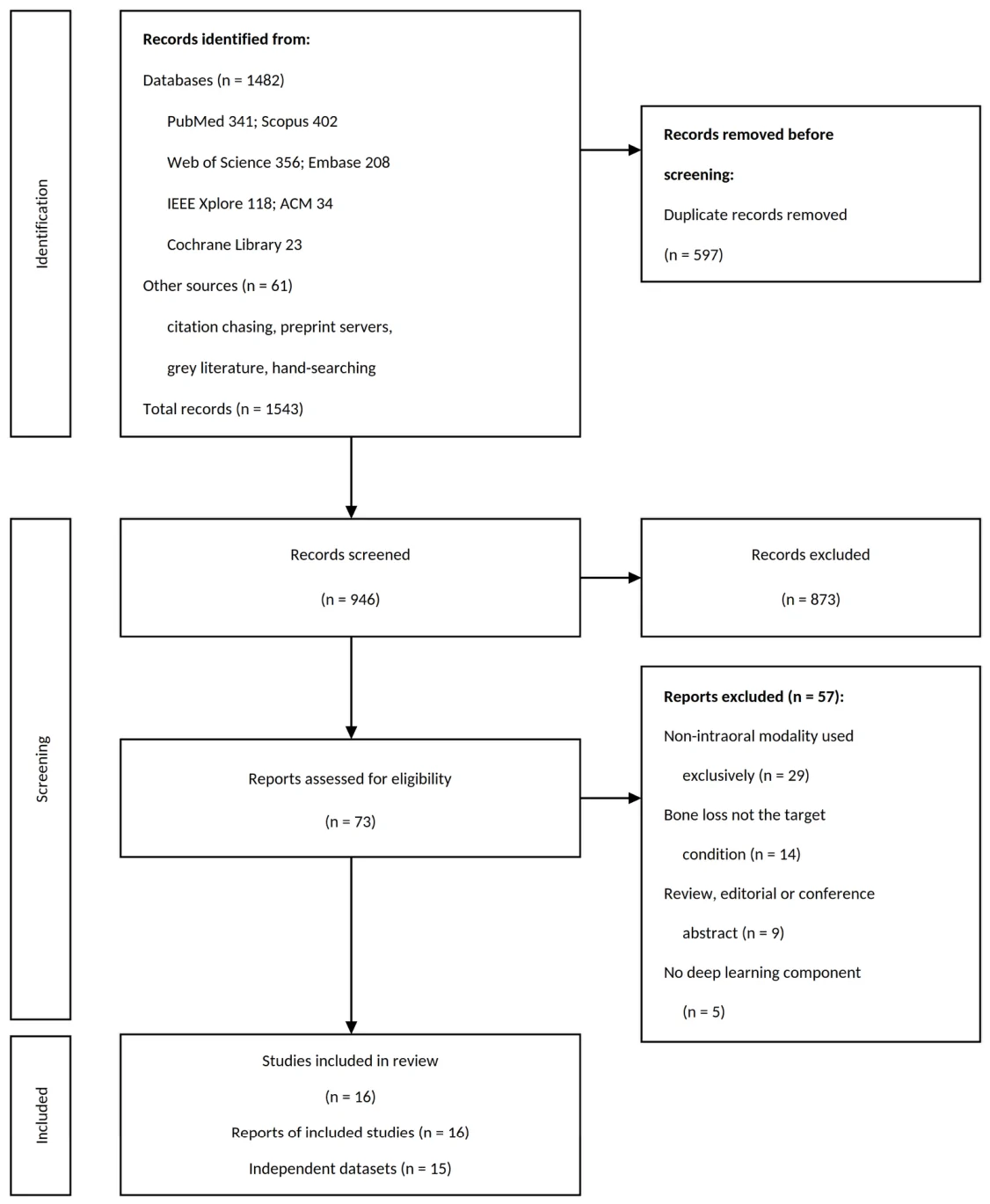
PRISMA 2020 flow diagram of study identification, screening and inclusion.

All records were imported into EndNote 21 and duplicates removed using a two-pass algorithm (automatic matching on DOI and on title–year–first author, followed by manual verification). Two reviewers independently screened titles and abstracts against the eligibility criteria in Rayyan, blinded to each other’s decisions. Records judged potentially eligible by either reviewer proceeded to full-text assessment. Full texts were then independently assessed by the same two reviewers, with reasons for exclusion recorded against a predefined hierarchy (wrong imaging modality > wrong target condition > wrong method > wrong publication type > no extractable outcome). Disagreements at either stage were resolved by discussion and, where consensus was not reached, by adjudication from a third reviewer. Inter-reviewer agreement was quantified using Cohen’s kappa at both stages, and the study selection process is presented as a PRISMA 2020 flow diagram (Figure 1). At revision, every excluded full-text report was re-verified against its full text and reassigned under this hierarchy, and every retained report was re-checked against the inclusion criteria; the outcome of that audit is reported in Section 3.1 and Supplementary File S5.

Screening was piloted on a random sample of 50 records before full screening began, with the eligibility criteria clarified and the reviewers recalibrated on the basis of the disagreements observed. No automation tool was used to make any inclusion or exclusion decision. Reports of the same dataset and the same model appearing in more than one publication were treated as duplicate reports rather than independent studies, and the most complete peer-reviewed version was retained. Where two reports analysed the same dataset but evaluated materially different models, both were retained and counted as a single dataset contributing two model comparisons; this rule was specified before screening and is applied in Section 3.1.

### 2.6. Data Extraction

A standardised data extraction form was developed in Microsoft Excel, piloted independently on five included studies and refined before full extraction. Two reviewers independently extracted data in duplicate; discrepancies were resolved by consensus. Where data were missing, ambiguous or reported only graphically, corresponding authors were contacted by email on two occasions two weeks apart; where no reply was received, the item was recorded as “not reported” and no imputation was undertaken. Where a study reported more than one model, test set, threshold or performance measure, four prespecified rules determined which value entered the synthesis. First, where several architectures were trained on the same data, the model designated by the study authors as their final or best-performing model on the independent test set was extracted as the primary result, and the full range across the architectures compared within that study is reported alongside it in Table 2, so that no within-study comparison is concealed by the choice. Second, where both internal and external test results were reported, the external result was extracted as primary, and the internal result reported next to it. Third, where several operating thresholds or cut-offs were reported, the threshold nominated by the authors as their primary operating point was used; where none was nominated, the threshold corresponding to the headline metric in the abstract of the source article was used and the choice recorded on the extraction form. Fourth, no training-set or validation-set figure was extracted as a performance result under any circumstances. Where a metric was presented only in graphical form and the authors did not respond, the value was recorded as not reported and that study contributed no value to the range for that metric; no value was estimated by reading off a figure, and no study was excluded from the review on this ground, so that the affected studies continue to contribute to every other outcome for which they report numerical results. The following domains were extracted:

- Bibliographic: first author, year, country, journal, study design, funding source, declared conflicts of interest, and commercial involvement.
- Dataset: number of radiographs, number of teeth or surfaces, number of patients, imaging projection (periapical, bitewing or mixed), sensor type and manufacturer, image resolution, single- versus multi-centre origin, inclusion/exclusion criteria for images, data augmentation, and whether the train/validation/test split was performed at patient level or image level.
- Reference standard: number, discipline and experience of annotators, calibration procedure, annotation software, method of resolving disagreement (consensus, majority vote, senior adjudication), reported intra- and inter-examiner reliability, and whether clinical periodontal data (CAL, probing depth) informed the ground truth.
- Model: architecture family and specific network, backbone, pretraining (e.g., ImageNet transfer learning), input dimensions, loss function, optimiser, learning rate, epochs, batch size, hardware, framework, hyperparameter tuning strategy, cross-validation scheme, and use of explainability methods (Grad-CAM, Eigen-CAM, saliency mapping).
- Task definition: binary detection of bone loss; multiclass severity or stage classification; continuous measurement of bone level or percentage bone loss; segmentation of anatomical structures; bone loss pattern classification (horizontal versus angular/vertical); furcation involvement detection.
- Outcomes: all reported performance metrics with confidence intervals where available; performance stratified by tooth type, region or stage where reported; direct comparison with human readers; and processing time.
- Transparency: availability of code, availability of the dataset, ethical approval, and registration of the study.

### 2.7. Risk of Bias and Methodological Quality Assessment

Risk of bias was assessed independently in duplicate using the Quality Assessment of Diagnostic Accuracy Studies–2 (QUADAS-2) tool [40], tailored to artificial intelligence diagnostic studies by the addition of signalling questions addressing (i) whether the test set was independent of the training and validation sets at the patient level; (ii) whether an external or multicentre test set was used; (iii) whether annotators were blinded to model output and to clinical data; (iv) whether images were consecutively or randomly sampled rather than selected for quality; and (v) whether the sample size was justified. Each of the four QUADAS-2 domains (patient selection, index test, reference standard, and flow and timing) was rated as low, high or unclear risk of bias, with applicability concerns rated for the first three domains. Decision rules for the additional questions were fixed before assessment began and are reported with the per-study answers in Supplementary File S3. An unfavourable answer to (i), a test set not independent of the training data at the patient level, or to (iii), where annotators were not blinded to the model output, was treated as sufficient on its own for a high risk-of-bias rating in the flow-and-timing and reference-standard domains, respectively, because each biases the accuracy estimate directly rather than conditionally. Unfavourable answers to (ii), (iv) and (v) did not by themselves produce a high-risk rating but were weighed together with the standard QUADAS-2 signalling questions for their domain. For (iv), consecutive or random sampling was required for a low-risk rating in the patient selection domain, because the review question concerns performance under routine conditions: selection of images on grounds of diagnostic quality was rated high risk where the filter was applied to the evaluation set, and unclear where the criteria or the number of images removed were not stated. Exclusion of images that were technically unusable, for example through gross exposure or processing failure, was accepted without penalty, since such images would be retaken in practice and do not narrow the clinical spectrum. A domain was rated unclear only where the report gave too little information to answer its signalling questions, never where the answer was unfavourable.

Completeness of reporting was assessed against the Checklist for Artificial Intelligence in Medical Imaging (CLAIM), 2020 version [41], with each of the 42 items scored as reported, partially reported or not reported, and an adherence percentage calculated per study. The 2020 version was used rather than the 2024 update because it was the version specified when the review was designed and the version against which the published dental artificial-intelligence appraisals used for comparison were scored, so that adherence percentages remain comparable. The update reorganises and expands the checklist to 44 items but does not alter the reporting domains covered; no included study was appraised under both versions, and rescoring the set under the update would not change the four items found to be unreported in every study. Overall methodological quality was additionally scored using APPRAISE-AI [42], which yields a 0–100 score across six domains (clinical relevance, data quality, methodological conduct, robustness of results, reporting quality, and reproducibility), with studies categorised as very low (<40), low (40–49), intermediate (50–59), high (60–79) or very high (≥80) quality. Where studies were compared with human readers, the reporting of that comparison was checked against STARD 2015 [43].

### 2.8. Outcomes and Effect Measures

The primary outcome is the diagnostic performance of deep learning models in assessing alveolar bone loss on intraoral radiographs, evaluated against a defined reference standard. Because the included studies frame the diagnostic problem in materially different ways, performance was extracted and synthesised separately for each task definition rather than aggregated across them. Six task definitions were prespecified.

- Binary detection: correct identification of the presence or absence of alveolar bone loss at the level of the image, tooth, interproximal surface or site. Effect measures: accuracy, sensitivity (recall), specificity, positive and negative predictive value, F1-score and area under the receiver operating characteristic curve, each with 95% confidence intervals where reported.
- Quantitative measurement of bone level: agreement between model-derived and reference measurements of the cemento-enamel junction to alveolar crest distance in millimetres, or of percentage bone loss relative to root length. Effect measures: intraclass correlation coefficient, Pearson or Spearman correlation, mean absolute error, limits of agreement, and formal tests of difference between model and examiner measurement.
- Multiclass stage assignment: correct assignment of radiographic bone loss stage or ordinal severity category. Effect measures: overall and per-class accuracy, per-class area under the curve, weighted kappa, and the full confusion matrix where reported, so that misclassification at adjacent-stage boundaries could be examined separately from overall error.
- Bone loss pattern classification: correct classification of the pattern as horizontal or angular/vertical. Effect measures: accuracy, sensitivity, specificity and F1-score.
- Furcation involvement detection: effect measures of precision, recall and F1-score, extracted and reported separately from aggregate bone loss performance rather than absorbed into it.
- Anatomical segmentation, where segmentation of bone area, tooth or cemento-enamel junction formed an intermediate step in a measurement pipeline: Dice similarity coefficient and intersection over union, reported per structure.

Disease prevalence in the test set was extracted alongside every accuracy figure. This was treated as a mandatory extraction field rather than an optional one, because accuracy and predictive values are prevalence-dependent and two models with identical discriminative ability can report substantially different accuracies purely as a function of case mix.

Secondary outcomes were: direct comparison of model performance against independent human readers evaluated on the same test set, including any formal statistical test of difference; model reproducibility on repeat presentation of identical images, compared against intra-examiner agreement; processing or reading time for model versus clinician; and performance stratified by radiographic projection, by tooth type and region, and by stage. Because these outcomes derive from single-timepoint diagnostic accuracy studies rather than longitudinal designs, no follow-up time points apply. Performance was extracted for every reported data partition, with the independent test set treated as the primary source and training or validation performance recorded separately so that the two could not be conflated.

### 2.9. Data Synthesis

A quantitative meta-analysis was planned a priori but was not undertaken, for reasons documented transparently here. Clinical heterogeneity was substantial: the target condition varied from binary presence of bone loss, through four-level stage assignment, to continuous percentage measurement and pattern classification, and the unit of analysis varied between the image, the tooth and the interproximal surface. Methodological heterogeneity was equally marked: reference standards ranged from single-reader annotation to three-reader consensus to clinically anchored diagnosis, and threshold definitions for “bone loss” differed between studies. Statistical heterogeneity could not be meaningfully quantified because most studies reported point estimates without confidence intervals and did not present the two-by-two contingency tables required for bivariate hierarchical modelling. Pooling under these conditions would have produced a spuriously precise summary estimate. Findings were therefore synthesised narratively following the Synthesis Without Meta-analysis (SWiM) reporting guidance [44], grouped by task type and by radiographic projection, and presented as structured tables with medians and ranges. Where three or more studies reported the same metric for the same task on the same unit of analysis, the median and interquartile range were computed descriptively and are explicitly labelled as such rather than as a pooled estimate.

Synthesis was ordered and interpreted in the light of risk of bias, with studies at low risk given greater weight in the narrative and the dependence of reported performance on methodological quality examined directly, in particular the relationship between reported accuracy and patient-level versus image-level data partitioning, test set prevalence, and the permissiveness of the threshold used to define the target condition.

Meta-analysis was prespecified as conditional rather than excluded outright. It would have been undertaken had a sufficiently homogeneous subset of studies been identified, defined a priori as sharing the same target condition, the same unit of analysis, a comparable threshold definition and extractable two-by-two contingency tables. Had those conditions been met, a bivariate random-effects model would have been fitted to estimate summary sensitivity and specificity with 95% confidence and prediction regions, a hierarchical summary receiver operating characteristic curve constructed, and heterogeneity assessed by inspection of forest plots and of the confidence and prediction regions rather than by I-squared, which is not appropriate for bivariate diagnostic accuracy models. Analyses would have been conducted in R using the mada and meta packages. The conditions were not met, and the specific reasons are reported in full above rather than summarised as heterogeneity.

Subgroup analyses were planned, where data permitted, by radiographic projection, by architecture family, by dataset size, by data split level, and by whether external validation was performed. Sensitivity analyses were planned excluding studies at high risk of bias in the patient selection or reference standard domains, and studies that did not confirm patient-level data partitioning. Where the number of contributing studies was too small for a subgroup or sensitivity analysis to be informative, this is stated rather than the analysis being presented on an inadequate base.

### 2.10. Certainty of Evidence

Certainty of evidence for each outcome was appraised using the GRADE approach adapted for diagnostic test accuracy [45], in which evidence from cross-sectional or cohort accuracy studies begins as high certainty and may be downgraded for risk of bias, indirectness, inconsistency, imprecision and publication bias, and upgraded for a very large effect. Publication bias was assessed qualitatively; funnel plot asymmetry testing was not appropriate given the absence of pooling and the small number of studies per outcome. Two reviewers rated certainty independently, and disagreements were resolved by consensus.

Certainty was rated separately for each outcome rather than for the review as a whole. This was prespecified because the diagnostic tasks under review differ substantially in difficulty and in the volume of supporting evidence, and a single aggregate rating would have flattened that gradient. Ratings were generated for six outcomes: binary detection of alveolar bone loss; quantitative measurement of bone level or percentage radiographic bone loss; multiclass periodontitis stage assignment; classification of bone loss pattern as horizontal or angular; detection of furcation involvement; and reading time or efficiency relative to human assessment. Certainty assessment was supported by the three appraisal instruments described in Section 2.7, and the summary of findings table states, for each outcome, the number of contributing studies, the range of reported performance, the certainty rating and the specific reasons for any downgrading.

### 2.11. Protocol Deviations

Four deviations from the registered protocol are declared. First, the planned bivariate random-effects meta-analysis was replaced by structured narrative synthesis, for the reasons set out in Section 2.9. Second, APPRAISE-AI was added post hoc as a supplementary quality instrument, because QUADAS-2 and CLAIM together do not capture the reproducibility and data quality domains specific to artificial intelligence research. Third, the registered record names Cochrane RoB-2, QUADAS-2 and ROBINS-I as the planned risk-of-bias instruments. RoB-2 and ROBINS-I are designed for randomised and non-randomised studies of interventions and are not applicable to diagnostic test accuracy or model-development studies; neither was applied. Risk of bias was assessed with QUADAS-2 supplemented by artificial-intelligence-specific signalling questions, as set out in Section 2.7. Fourth, the registered record names seven information sources; because registration was retrospective, that list does not include three sources that had already been searched—IEEE Xplore, the ACM Digital Library and the Cochrane Central Register of Controlled Trials—which were used to capture the computer-science and medical-imaging proceedings in which dental artificial intelligence work frequently appears first. Every source named on the record was searched: MEDLINE via PubMed, Scopus, Embase, the Science Citation Index Expanded within the Web of Science Core Collection, Google Scholar and ClinicalTrials.gov; no registered source was omitted. The complete set of seven databases and six supplementary sources is given in Section 2.4. No change was made to the eligibility criteria or to the outcome definitions after registration. The registered record declares the overlap with three previously registered reviews described in Section 2.1 and states the grounds on which the present review is distinguished from them.

## 3. Results

The electronic searches yielded 1482 records (PubMed 341, Scopus 402, Web of Science 356, Embase 208, IEEE Xplore 118, ACM Digital Library 34, Cochrane Library 23), and a further 61 records were identified from citation chasing, preprint servers, grey literature and hand-searching. After removal of 597 duplicates, 946 records were screened by title and abstract, of which 873 were excluded as clearly irrelevant, non-radiographic, non-dental or of ineligible publication type; inter-reviewer agreement at this stage was substantial (Cohen’s κ = 0.86). Seventy-three full-text articles were assessed for eligibility, and 57 were excluded with reasons: panoramic, cone-beam computed tomography or other non-intraoral radiographic input used exclusively (*n* = 29), bone loss not the target condition (*n* = 14), review, editorial or conference abstract (*n* = 9), and no deep learning component (*n* = 5). No report was excluded solely for want of an extractable performance metric or as a duplicate publication of an included dataset; those two categories are therefore empty. The same audit recovered two reports that had been assessed at full text and are discussed in this Section but had been omitted from the excluded-study list; the number of reports assessed for eligibility is therefore 73 rather than 71, and the number excluded at title and abstract is 873 rather than 875. Inter-reviewer agreement at full-text stage was almost perfect (κ = 0.91). Sixteen publications, reporting 15 independent datasets, were included in the qualitative synthesis. During the revision of this review, two reports initially retained were re-examined against their source articles and reclassified as ineligible. The first was found to have used 640 panoramic radiographs exclusively; periapical and bitewing images appear only in its background and reference list [46]. That report was therefore reclassified as ineligible under the modality criterion. The second evaluates tooth-number recognition and full-mouth-series arrangement rather than alveolar bone loss; the bone and cemento-enamel junction masks it generates serve tooth localisation, its reported outcomes are charting metrics, and the source article names alveolar bone assessment as a prospective module rather than an evaluated output. It was therefore reclassified under the wrong-target-condition criterion [47]. Both reports are listed among the excluded full-text articles in Supplementary File S5, and all counts, tables and figures in this review reflect the corrected set of 16 included publications. The selection process is summarised in Figure 1, and the full list of excluded full-text reports with reasons is provided as Supplementary File S5.

The largest exclusion category warrants comment, because several of the excluded reports are frequently cited as evidence for deep learning in periodontal diagnosis. Studies restricted to panoramic radiography (including the foundational work of Krois and colleagues and the DeNTNet transfer network, and subsequent hybrid, two-stage, ensemble and segmentation approaches [18,19,48,49,50,51,52,53]) were excluded under the modality criterion notwithstanding their methodological quality, for the reasons developed in Section 4.2. Studies applying classical machine learning to clinician-measured numerical features, or texture-based methods such as fractal dimension analysis, rather than learning representations from image data, were excluded under the method criterion [54].

### 3.1. Characteristics of the Included Studies

The sixteen included publications were published between 2018 and 2026, with a marked acceleration after 2021: one study appeared before 2021, ten between 2021 and 2023, and five from 2024 onwards. Geographically, the evidence base is concentrated in East Asia (Republic of Korea, Taiwan and China, n = 4), Türkiye (n = 3), the United States (n = 2) and Germany (n = 2), with single studies from the United Kingdom, the Netherlands, Brazil, Saudi Arabia and Pakistan. All were retrospective analyses of archived radiographic datasets; none was a prospective diagnostic accuracy study, and none evaluated clinical impact. Dataset sizes varied by nearly three orders of magnitude, from 39 radiographs in a preliminary evaluation of a commercially cleared system to 21,819 radiographs in the largest series. Eleven publications used periapical radiographs exclusively, two used bitewing radiographs exclusively, and three used mixed periapical and bitewing datasets. One pair of publications reports overlapping data: Dujic and Hoss analysed the same institutional dataset of 21,819 periapical radiographs using different architecture families, and that pair is counted once wherever the number of independent datasets is relevant. Sixteen publications therefore contribute 15 independent datasets. Study characteristics are summarised in Table 3.

Two of the included reports (Dujic and colleagues and Hoss and colleagues [33,34]) analyse the same institutional dataset of 21,819 periapical radiographs but evaluate different architecture families, five vision transformer networks in the former and five convolutional networks in the latter. They were therefore retained as companion reports rather than excluded as duplicate publications, and their findings are treated as one dataset with two architectural comparisons wherever a count of independent datasets is relevant. No other pair of included studies shares a dataset.

Reference standards were classified into mutually exclusive categories and are tabulated for every included publication in Supplementary File S7. Seven publications used multi-examiner consensus; two used manual landmark or distance measurement; one used majority voting; two used single-examiner radiographic annotation; one used radiographic assessment anchored to clinical attachment level; one used multi-examiner annotation without a stated resolution rule; and three described the annotation procedure too briefly to permit classification. No included publication used a three-dimensional, surgical or histological reference standard, so in every case the ground truth was expert interpretation of the same two-dimensional radiograph on which the model was evaluated. Inter- or intra-examiner agreement was reported as a statistic by only three publications, and no publication reported whether annotators were blinded to clinical information or to model output.

### 3.2. Deep Learning Architectures and Training Strategies

Four architectural generations were discernible across the included studies, broadly tracking developments in the wider computer vision literature (Table 4).

#### 3.2.1. Classification Convolutional Neural Networks

The earliest studies framed the problem as whole-image or cropped-region classification. Lee and colleagues combined a pretrained deep CNN architecture with a self-trained network to classify periapical images of premolars and molars as periodontally compromised or not, reporting diagnostic accuracy of 81.0% for premolars and 76.7% for molars, and accuracy of 82.8% (95% CI 70.1–91.2) and 73.4% (95% CI 59.9–84.0) respectively for predicting the need for extraction in severely compromised teeth [24]. Alotaibi and co-workers applied VGG-16 to 1724 periapical images of anterior teeth and found the network able both to detect bone loss and to categorise its severity with satisfactory performance, while noting reduced discrimination between adjacent severity classes [29]. This generation is computationally light and easy to train, but it returns only a label; it provides no localisation, no measurement, and no explanation, and it is highly sensitive to how the region of interest is cropped.

#### 3.2.2. Encoder–Decoder Segmentation Networks

The second generation reframed the task as pixel-level segmentation of the anatomical structures from which bone loss is derived. The most influential example is the pipeline reported by Lee and colleagues, in which three separate segmentation networks (for bone area, tooth and CEJ) were integrated with a geometric image-analysis module to compute the percentage of RBL, assign an RBL stage, and generate a provisional periodontal diagnosis under the 2018 classification [30]. The average Dice similarity coefficient for segmentation exceeded 0.91; there was no statistically significant difference between the percentage RBL measurements produced by the model and those produced by independent examiners (*p* = 0.65); the areas under the receiver operating characteristic curve for stage I, II and III assignment were 0.89, 0.90 and 0.90 respectively; and the accuracy of case-level diagnosis was 0.85. The automated analysis took approximately nine minutes compared with 137 min for the clinicians, a fifteen-fold difference obtained in a single study under defined experimental conditions. This architecture is conceptually attractive because it reproduces the measurement the clinician actually makes, rather than substituting a black-box label for it, and because its intermediate outputs are directly inspectable.

#### 3.2.3. Object Detectors and Keypoint Localisers

A third strategy localises the specific anatomical landmarks required for measurement. Danks and colleagues trained a landmark-localisation network to identify the CEJ, the alveolar crest and the root apex on periapical radiographs, and derived the percentage bone loss geometrically from those coordinates [26]. Chen and co-workers subsequently developed an ensemble CNN that simultaneously predicted tooth position, detected tooth shape, identified the remaining interproximal bone level and derived RBL from periapical and bitewing radiographs, an approach that yields an auditable measurement rather than a categorical label [32].

#### 3.2.4. Vision Transformers and Hybrid Ensembles

The most recent studies have moved beyond convolutional inductive biases. A single institutional dataset of 21,819 anonymised periapical radiographs from anterior and posterior, maxillary and mandibular regions, graded by calibrated dentists into four categories (no bone loss; mild, under 15% of root length; moderate, 15–33%; and severe, beyond the coronal third), has been used to benchmark both architecture families. Dujic and colleagues evaluated five vision transformer networks (ViT-base, ViT-large, BEiT-base, BEiT-large and DeiT-base) for automated detection of periodontal bone loss [33], while Hoss and colleagues evaluated five convolutional networks on the same images [34], with transformer architectures proving competitive with, and in places superior to, their convolutional counterparts. This remains, by a considerable margin, the largest intraoral dataset in the field, and the paired design makes it the only direct architecture-family comparison available on identical data. In parallel, segmentation-based multi-class detectors have been used to produce complete radiographic charts: Bayırlı and colleagues trained a YOLOv8x-seg model for 800 epochs with extensive augmentation on patient-level splits to detect alveolar bone loss, calculus and furcation defects alongside restorative conditions on bitewing radiographs, achieving the highest performance for the alveolar bone loss class (precision 0.84, recall 0.93, F1 0.88) but markedly weaker performance for low-prevalence classes such as furcation defect (F1 0.40) and cervical marginal gap (F1 0.23) [39].

#### 3.2.5. Explainability

Only a minority of the included studies incorporated any interpretability mechanism. Sunnetci and colleagues used Eigen-CAM mapping to visualise the image regions driving four-class staging decisions on 1752 bitewing radiographs, in which lesions were categorised as healthy, stage I (under 15% bone loss), stage II (15–33%) and stage III–IV (over 33% with furcation destruction) [37]. Others applied Grad-CAM to periapical classification models. Where explainability was reported, activation maps generally localised to the interproximal crestal region, which is reassuring, but no study formally evaluated whether the explanations were faithful, nor whether they changed clinician trust or behaviour.

### 3.3. Diagnostic Performance

#### 3.3.1. Binary Detection of Bone Loss

Eleven studies reported performance for the binary task of distinguishing sites, teeth or images with bone loss from those without. Comparative statements made across studies in this subsection are based on accuracy, the only metric reported by all eleven; sensitivity and specificity are given where the source reports them, and are not used to rank studies because they are not available for the whole set. Reported accuracy spanned approximately 0.73 to 0.97, with the median in the region of 0.81–0.84. Sensitivity was generally reported between 0.65 and 0.93 and specificity between 0.66 and 0.99; the lowest specificities were recorded by the two transformer and convolutional network comparisons on the largest single dataset [24,29,30,33,34,38,39]. The highest values came from studies with the largest datasets and the cleanest binary definitions; the lowest came from studies with small datasets, unselected image quality, or anatomically difficult regions. Two features of this distribution deserve emphasis. First, the range is wide enough that a single headline figure would be misleading. Second, accuracy correlated more closely with how permissively the target condition was defined than with the sophistication of the architecture: studies that dichotomised at a low threshold (any detectable crestal bone loss) reported higher accuracy than studies that dichotomised at a clinically meaningful threshold aligned to stage II or above.

#### 3.3.2. Quantitative Measurement of Bone Level

Five studies reported continuous measurement of bone level or percentage bone loss rather than categorical classification. This task outputs the quantity used directly in stage assignment, the percentage of root length affected; its clinical interpretation is considered in Section 4.8. Lee and colleagues found no statistically significant difference between DL-derived and examiner-derived percentage RBL (*p* = 0.65), with segmentation Dice coefficients above 0.91 [30]. Danks and colleagues reported landmark localisation errors that the authors judged to fall within the tolerance they prespecified for that measurement, and derived percentage bone loss with good agreement [26]. In the only evaluation of a regulator-cleared commercial system, mean absolute error against calibrated examiners was 0.499 mm on bitewing surfaces [38]. Reported intraclass correlation coefficients between model and expert measurement clustered between 0.75 and 0.85, and mean absolute errors were typically in the range of 0.3–0.9 mm or 4–8 percentage points. These figures are of the same order as published inter-examiner error for manual measurement; the interpretation of that comparison is developed in Section 4.1.

#### 3.3.3. Multiclass Staging

Seven studies attempted assignment to periodontitis stage or to an ordinal severity category. Performance was consistently lower than for binary detection, and the loss was concentrated at the boundaries between adjacent classes, most often between stage I and stage II, and between stage III and stage IV. Lee and colleagues reported per-stage AUCs of 0.89, 0.90 and 0.90 for stages I, II and III, and a case-level diagnostic accuracy of 0.85 [30]. Erturk and colleagues, working with four classes on 1752 bitewing images, likewise reported strong discrimination between healthy and severe categories but substantially weaker separation of the intermediate stages [37]. This is diagnostically consequential, because the stage I/II and stage III/IV boundaries influence treatment complexity and referral decisions, and because misclassification across them carries different implications from misclassification within them. They do not, however, determine management on their own. Staging under the 2018 classification integrates radiographic bone loss with interdental clinical attachment level, probing depth and bleeding on probing, furcation involvement, tooth mobility and prognosis, tooth loss attributable to periodontitis, masticatory function, patient-level risk factors and the response to initial therapy. Automated radiographic staging should therefore be understood as contributing one component of this composite assessment, informing rather than replacing the clinician’s judgement about whether non-surgical therapy, surgical referral, or extraction and rehabilitation is appropriate.

#### 3.3.4. Bone Loss Pattern and Furcation Involvement

Only two included studies addressed the pattern of bone loss (horizontal versus angular/vertical) or furcation involvement directly, and this is the sparsest area of the evidence base. Where the pattern was derived geometrically from segmented bone and tooth masks rather than classified as a label, performance was acceptable, but no study validated pattern classification against surgical or three-dimensional confirmation. Furcation detection was the weakest-performing task across the entire review: in the multi-class bitewing charting study, the furcation defect class achieved an F1 score of only 0.40, against 0.88 for alveolar bone loss in the same model [39]. Two explanations are plausible and not mutually exclusive: furcation involvement is intrinsically difficult to visualise on two-dimensional images because of superimposition of roots and buccal or lingual plates, and furcation cases are comparatively rare in unselected datasets, so the class is severely under-represented during training. The practical implication is the same either way: furcation status should continue to be established clinically, and a negative model output should not be taken as evidence of absence.

#### 3.3.5. Comparison with Human Readers

Six studies compared model output directly with human readers evaluated on the same test set. The consistent finding was parity rather than superiority. Where models outperformed dentists numerically, the difference was rarely statistically significant; where they underperformed, the gap was small. What was uniformly in favour of the model was speed, although the evidence for this is narrower than it may appear. Only one included study reported timing data: in that study, automated analysis required approximately 9 min against approximately 137 min for clinicians on the same image set [30]. This difference derives from a single study conducted under defined experimental conditions, with no measure of variability and no replication in an independent setting, and it should not be read as an estimate of the time saved in routine clinical workflows. No other included publication reported a model-versus-clinician timing comparison, or a model inference time.

A related claim requires similar qualification. Deep learning models return an identical output when the same digital image is presented again, which demonstrates *deterministic repeatability* rather than reproducibility of measurement across different acquisitions, sensors, exposure parameters, projection geometries or institutions. No included study assessed repeat-acquisition or cross-institutional reproducibility, and the stability of model output under realistic variation in image acquisition therefore remains untested.

### 3.4. Risk of Bias and Methodological Quality

QUADAS-2 assessment revealed a consistent pattern (Table 5). The *patient selection* domain was at high or unclear risk of bias in the majority of studies, because images were retrospectively drawn from institutional archives with quality-based inclusion criteria (blurred, distorted, foreshortened or heavily restored images were commonly excluded), which produces a spectrum that is systematically easier than routine clinical practice and inflates apparent accuracy. The *index test* domain was generally at lower risk, since model outputs are deterministic and thresholds were usually prespecified, although several studies did not state whether the threshold was selected before or after examining test-set performance. The *reference standard* domain was the most conceptually problematic: in almost all studies the ground truth was itself a radiographic judgement made by human examiners, so the models were trained to reproduce human interpretation rather than to detect the underlying biological reality. Only a minority anchored the reference standard to clinical periodontal data, and none used cone-beam computed tomography or surgical/histological confirmation. The *flow and timing* domain was frequently at unclear risk because the relationship between the timing of the radiograph, the timing of the clinical examination and the timing of annotation was not described.

Two structural findings emerged from the signalling questions and are tabulated in full in Supplementary File S3. Only four of the sixteen studies explicitly confirmed that data partitions were disjoint at patient level; the remainder either split at image level or did not state the level at all. External validation on a dataset from a different institution, sensor or population was performed in two studies: an evaluation of a regulator-cleared commercial system, and one study that tested its segmentation networks on radiographs from separate institutions. No study reported prospective validation, and none measured any clinical outcome. The implications of these two findings are developed in Section 4.4.

CLAIM adherence ranged from 67% to 82% of applicable items, with a median of 77%. Four items were unreported in every included publication: the intended sample size and how it was determined; how missing data were handled; the registration number and registry; and where the full study protocol can be accessed. Validation or testing on external data was unreported in 14 of the 16 publications, and methods for explainability or interpretability in 11. Only three studies made code publicly available, and only one made an anonymised dataset available, which materially limits reproducibility. Under APPRAISE-AI, no study reached the very high-quality band (80–100). Eleven of the sixteen studies fell in the high band (60–79) and five in the intermediate band (40–59); none scored below 40. Total scores ranged from 50 to 72. Studies with declared commercial involvement warranted particular scrutiny, although the most recent of these implemented appropriate safeguards by excluding the commercially affiliated author from the annotation and adjudication process [39].

### 3.5. Certainty of the Evidence

Certainty was rated separately for each outcome (Table 6). For binary detection of alveolar bone loss, the certainty was rated very low, downgraded two levels for risk of bias (retrospective quality-filtered image selection, image-level data splitting, human-derived reference standards) and one level for inconsistency (accuracy ranging from 0.73 to 0.97 across studies), with no upgrade applied. For quantitative measurement of bone level, certainty was rated very low, downgraded two levels for risk of bias and one level for imprecision (few studies, small samples, confidence intervals rarely reported). For multiclass staging, certainty was rated very low, downgraded additionally for indirectness, since staging in the source studies was defined radiographically alone whereas the 2018 classification requires integration of radiographic and clinical data. For furcation and pattern classification, certainty was rated very low on the basis of very few studies, small class sizes and weak reported performance. For reading time and efficiency, certainty was rated very low: a single study provided a model-versus-clinician comparison, obtained under experimental rather than routine clinical conditions and without independent replication. Certainty was therefore very low for all six outcomes, consistent in each row with the cumulative number of downgrade levels applied.

## 4. Discussion

### 4.1. Principal Findings

This review synthesised sixteen publications, reporting 15 independent datasets, applying deep learning to the assessment of alveolar bone loss on intraoral radiographs. Three findings stand out. First, in the small number of studies that evaluated models and clinicians on the same test set, selected internally validated models achieved performance within the range observed for calibrated readers under controlled experimental conditions; this is not a general property of the evidence base, and no study performed a formal non-inferiority analysis. Second, performance degrades systematically as the task moves closer to the clinical decision: binary detection is easier than continuous measurement, which is easier than multiclass staging, which is easier than furcation and pattern characterisation. Third, and most importantly, the methodological quality of the evidence lags behind the technical performance being claimed. Apparently strong technical performance has not yet been matched by equally strong external validation, clinically anchored reference standards, or prospective evidence, and this constrains what can presently be concluded from it.

Throughout this Discussion we distinguish four levels of claim: findings reported directly by the included studies; patterns identified across the review as a whole; interpretations made by the review authors; and applications proposed for future evaluation. Statements in the last two categories are identified as such, and are not presented as demonstrated clinical benefits. In particular, longitudinal monitoring, triage, quality assurance, audit and the reduction in examiner drift are plausible future uses of this technology; no prospective implementation study evaluating any of them was identified by this review.

Because the measurement errors reported in Section 3.3.2 are of the same order as published inter-examiner error for manual measurement, the operative question raised by this evidence is not whether a model is accurate in absolute terms, which no annotation-based evaluation can establish, but whether it is at least as consistent as the examiners it would support. The evidence synthesised here indicates that deep learning models achieve high diagnostic performance for the radiographic assessment of alveolar bone loss, and that in several specific tasks their performance is comparable to, and in some reports exceeds, that of human readers of varying clinical experience. The clinical value of these systems is therefore unlikely to rest on any single dimension. It appears instead to lie in the combination of accurate image-based detection with deterministic measurement and the automation of repetitive quantification—a combination that could reduce the burden of routine image analysis and release clinician time for patient-specific reasoning and decision-making. Longitudinal periodontal monitoring, in which the question is whether bone level has changed since the previous radiograph, is particularly vulnerable to examiner drift and is therefore particularly well suited to automation.

Accurate performance on an image-based diagnostic task is nevertheless not equivalent to clinical decision-making. A radiographic finding acquires meaning only when it is integrated with the patient’s history, clinical examination, probing and attachment-level findings, risk factors, disease trajectory, available treatment options and individual circumstances. The consequences of a false-positive and a false-negative output differ materially for the patient, and questions of accountability, explainability, patient safety and legal and ethical responsibility constitute constraints on autonomous use that are independent of measured accuracy.

Human oversight is therefore required not because these systems lack diagnostic capability, but because their output must be interpreted within a wider clinical context and the final decision must remain with a responsible clinician. On the present evidence, deep learning systems for the assessment of alveolar bone loss on intraoral radiographs are best positioned as clinician-supervised adjuncts that support diagnosis and measurement, rather than as independent clinical decision-makers.

### 4.2. Why the Intraoral Restriction Matters

Restricting this review to intraoral radiography was a deliberate methodological choice. Periapical and bitewing projections differ from panoramic radiography in spatial resolution, geometric distortion, visibility of the CEJ and role in periodontal staging, and pooling them would make performance estimates difficult to interpret. The restriction also matches clinical practice: intraoral imaging is the projection on which definitive staging decisions are made. Applying the criterion consistently required excluding one report that had been retained in error, as described in Section 3.1.

### 4.3. The Reference Standard Problem

The most fundamental limitation of this literature is epistemic rather than technical. In almost every included study, the ground truth against which the model was evaluated was the annotation of one to three human examiners looking at the same two-dimensional image. The model is therefore optimised to reproduce human radiographic interpretation, including its systematic errors. It is well established that two-dimensional radiography underestimates bone loss relative to cone-beam computed tomography, particularly for buccal and lingual defects and for intrabony components, and that examiners disagree substantially in the furcation region [9,11]. A model achieving 0.95 agreement with a human annotator has demonstrated fidelity to that annotator, not accuracy against the periodontium. The scale of this constraint is visible in the largest dataset in the field, where the inter-examiner Cohen’s kappa for the four-category bone loss reference standard was reported as 0.454 to 0.482, with intra-examiner agreement of 0.739; on the conventional interpretation, these represent moderate rather than substantial agreement, and they bound what any model trained on that standard can be shown to achieve. The influence of that ceiling can be attenuated but not removed. Large, heterogeneous, multicentre datasets annotated by several examiners can dilute the idiosyncratic and random components of annotator error, since a model trained across diverse samples learns recurring anatomical patterns rather than the habits of one reader, and multiple independent annotations, consensus review and the explicit reporting of annotation uncertainty act in the same direction. Annotation-based evaluation can also work against the model: an output that segments or localises correctly but departs from the original annotation is scored as an error, so reported performance may understate true performance, a recognised limitation of this form of assessment. What none of these measures can do is establish that a model is more accurate than the annotators who defined its reference standard, because the comparison has no independent arbiter; systematic error shared by all annotators, such as the underestimation of bone loss inherent to two-dimensional projection, is reproduced by the model however large or diverse the dataset. Data diversity, multiple expert annotation, consensus adjudication, reporting of annotation uncertainty and clinically anchored reference standards are therefore complementary requirements rather than alternatives to one another. Future studies should accordingly combine them, anchoring ground truth to clinical attachment level measurements, to surgical exposure where ethically available, or to cone-beam computed tomography in the subset of patients in whom it is clinically indicated for other reasons.

A related and rarely discussed issue is that the 2018 classification defines periodontitis stage and grade using an integration of radiographic bone loss, clinical attachment level, tooth loss attributable to periodontitis, probing depths, furcation status, defect morphology, and the rate of progression relative to age and risk factors [3,4]. No radiograph, and therefore no model reading a radiograph, can supply that full dataset. Studies that describe their output as a “periodontitis stage” are, strictly, producing a radiographic bone loss stage that is one input to staging. The distinction is not pedantic: it determines whether the technology is positioned as a decision-support component within a clinician-led diagnostic process, which is defensible, or as an autonomous diagnostic device, which the evidence does not support.

### 4.4. Dataset Limitations, Generalisability and Data Leakage

The included datasets were small by the standards of medical imaging and were drawn predominantly from single institutions. Quality-based image exclusion was near-universal, producing a spectrum systematically easier than routine practice and reducing applicability to it. Several studies partitioned data at image rather than patient level, so images from the same patient could appear in both training and test sets; where clustering is ignored, confidence intervals are too narrow, and precision is overstated. External validation was reported by only two studies.

Image-level rather than patient-level data splitting was the single most common methodological flaw. When a full-mouth series from one patient is randomly divided between training and test sets, the test set is no longer independent: the model has seen that patient’s anatomy, restorations and bone morphology, and reported accuracy is optimistically biased. Only four of the sixteen publications confirmed patient-level splitting. Reviewers and editors should treat an unstated split level as a red flag, and future studies should report it explicitly as a mandatory item.

Class imbalance compounds the problem. Datasets drawn from periodontal clinics over-represent disease; datasets drawn from general practice under-represent severe disease and furcation involvement. Because accuracy is prevalence-dependent, two models with identical discriminative ability can report very different accuracies simply because their test sets had different disease prevalence. This is the most likely explanation for the wide accuracy range observed across the included studies, and it is a strong argument for reporting sensitivity, specificity, predictive values and prevalence separately rather than headline accuracy alone.

### 4.5. Explainability, Trust and Clinical Integration

The minority of studies that implemented Grad-CAM or Eigen-CAM found that model attention localised to the interproximal crestal region, which is anatomically plausible [37,55]. However, class activation mapping demonstrates where a model looked, not why it decided, and post hoc saliency methods have been shown in other domains to be unstable and sometimes uninformative [56]. From a clinical governance perspective, segmentation-based and landmark-based pipelines have an inherent advantage over classification networks: their intermediate outputs (the bone outline, the CEJ position, the measured distance) are the same quantities the clinician would measure, and can therefore be visually verified and manually corrected. Architectures that expose an auditable measurement rather than an opaque label are likely to be adopted more readily, and are more defensible medico-legally.

Human–AI interaction has been almost entirely neglected. No included study examined whether providing model output to a clinician improved that clinician’s diagnostic accuracy, changed treatment planning, altered referral patterns, or induced automation bias, the well-documented tendency to accept an incorrect machine recommendation [57]. Since the realistic deployment scenario is clinician-plus-model rather than model-alone, this is the most consequential evidence gap in the field.

### 4.6. Comparison with Previous Reviews

Previous syntheses have generally combined panoramic and intraoral studies, reporting pooled estimates across modalities that are difficult to interpret for either. Restricting the present review to intraoral imaging yields lower headline figures than those syntheses but figures that are anchored to a single, clinically coherent projection. Three further reviews of overlapping scope were registered in PROSPERO in May 2026; the present review differs in its restriction to intraoral radiography, its separation of results by diagnostic task and unit of analysis, and its use of three complementary appraisal instruments. Two syntheses are directly comparable with the present one. Li and colleagues pooled studies classifying periodontitis stage on dental images irrespective of projection and reported summary accuracy above the range observed here [20]. Their included set is dominated by panoramic datasets, in which the whole dentition is captured in a single image, annotation is performed at tooth or jaw level, and the effective unit of analysis is therefore coarser than the interproximal site used in most intraoral studies; the architectures are correspondingly weighted towards whole-image classification networks rather than the segmentation and landmark pipelines that predominate here. Alqutaibi and colleagues restricted their synthesis to two-dimensional radiographs and, like the present review, applied APPRAISE-AI; their appraisal reached the same conclusion on methodological quality, with the lowest scores concentrated in the same domains of data quality and reproducibility, but they combined panoramic with intraoral inputs and classical machine learning with deep learning, which widens the architecture range and admits reference standards defined at jaw rather than site level [21]. The earlier scoping review of Schwendicke and colleagues described the field before encoder–decoder segmentation and transformer architectures were adopted and before external validation had become an expectation of reporting, which accounts for most of the difference in the validation evidence available to it [16]. The divergence between these syntheses and the present review is therefore attributable less to disagreement about the primary studies than to four design choices that can be stated explicitly: the imaging modality admitted, which fixes resolution and unit of analysis; the architecture families admitted, which determines whether classical machine learning contributes to the pooled estimate; the validation evidence demanded, since earlier pooled estimates are dominated by internally validated models and fall when external validation is required; and the reference standard accepted. On the last of these, the reviews agree, and the agreement is the most robust finding available across them: no synthesis in this field, including this one, has identified a study anchored to a three-dimensional, surgical or histological standard, so the binding constraint on the literature is the reference standard rather than the model.

### 4.7. Strengths and Limitations of This Review

Strengths include a registered protocol; a comprehensive multi-database search extended to engineering databases, preprint servers and grey literature, which is essential in a field where much work is published outside the dental literature; duplicate independent screening, extraction and appraisal; the use of three complementary appraisal instruments (QUADAS-2, CLAIM and APPRAISE-AI) that together cover diagnostic accuracy bias, reporting completeness and artificial-intelligence-specific reproducibility; and transparent GRADE rating by outcome.

Limitations must equally be acknowledged. Restriction to English-language publications may have excluded relevant work, particularly from East Asia. Meta-analysis was not feasible, so no pooled estimate with confidence intervals is offered; the ranges presented are descriptive and should not be interpreted as summary accuracy. The boundary between “deep learning” and “classical machine learning” is not always crisp in hybrid pipelines, and eligibility decisions in a small number of borderline studies required judgement. Publication bias is very likely and could not be formally assessed: models that underperform are rarely published, and the near-total absence of reported negative results in this literature is itself evidence of selective reporting. Finally, the field is moving quickly enough that any synthesis has a short half-life; this review should be regarded as a living baseline rather than a settled account.

### 4.8. Implications for Practice

On the current evidence, deep learning applied to intraoral radiographs is suitable for use as an adjunct under clinician supervision in three scenarios: as a screening and flagging tool that draws attention to sites with probable bone loss in a full-mouth series; as a measurement aid that produces a first-pass percentage bone loss figure for clinician verification; and as a quality-assurance and audit instrument for retrospective review of radiographic diagnosis within a practice or teaching institution. It is not suitable for autonomous diagnosis, for autonomous stage assignment, or for treatment planning decisions without clinician review. Any clinical deployment should retain the radiograph and the clinician’s own assessment as the record of record, should log model outputs for audit, and should be preceded by local validation on the institution’s own sensors and patient population.

### 4.9. Implications for Research

- Assemble and share multicentre, multi-sensor, demographically diverse intraoral radiographic datasets with standardised annotation protocols, and publish them openly so that models can be benchmarked against one another rather than against incomparable private datasets.
- Anchor reference standards to clinical data (clinical attachment level and probing depth) rather than to radiographic annotation alone, and report annotator number, experience, calibration procedure and inter-annotator agreement as mandatory items.
- Mandate patient-level data splitting and report it explicitly; report prevalence in the test set alongside all accuracy figures; and report confidence intervals for every performance estimate.
- Perform external validation on data from an institution, sensor and population not represented in training and report the performance drop honestly.
- Report performance stratified by tooth type, region, stage and pattern, and specifically report furcation performance separately rather than absorbing it into an aggregate figure.
- Prioritise architectures that expose auditable intermediate measurements over opaque classifiers, and evaluate explainability methods for faithfulness rather than merely presenting heat maps.
- Conduct prospective, ideally randomised, studies of clinician-plus-model versus clinician-alone, with diagnostic accuracy, treatment planning concordance, reading time, referral appropriateness and automation bias as endpoints.

Adhere prospectively to CLAIM, and to the CONSORT-AI and TRIPOD-AI/PROBAST-AI frameworks as these mature [58,59], and register model-development studies as one would register a clinical trial.

## 5. Conclusions

Deep learning models applied to intraoral radiographs can detect alveolar bone loss, segment the structures required for bone level measurement, and quantify percentage radiographic bone loss. Evidence is strongest for these simpler detection and segmentation tasks and weakest for multiclass stage assignment, pattern classification and furcation involvement; a high segmentation coefficient does not establish accurate periodontal staging. Where models and clinicians were evaluated on the same test set, selected internally validated models performed within the range observed for calibrated readers under controlled experimental conditions, and no formal non-inferiority comparison was reported. Substantial time savings were reported in selected experimental settings, but processing time depends on hardware, preprocessing, image quality, workflow integration and any manual review and is not evidence of improved efficiency in routine practice.

Certainty is very low for every diagnostic outcome assessed. Most studies are retrospective and single-centre; datasets are commonly filtered for image quality, which may produce a spectrum easier than routine practice and reduce applicability to it; data splitting is frequently at image rather than patient level; external validation is rare; and prospective clinical evaluation is absent. Reference standards are almost universally expert interpretation of the same two-dimensional images, so agreement with that reference demonstrates agreement with the annotators rather than diagnostic validity against the biological extent of periodontal destruction. These systems are therefore better described as potential clinician-supervised adjuncts for screening, measurement support and quality assurance than as autonomous diagnostic tools. Progress will depend less on architectural innovation than on methodological discipline: multicentre datasets, clinically anchored reference standards, patient-level validation, transparent reporting, and prospective studies that measure whether patients are better diagnosed and treated when the model is present.

## Figures and Tables

**Table 1:** PICO/PIRD framework, review questions and corresponding search concepts.

Element	Definition Applied in This Review	Core Search Concepts and Controlled Vocabulary
P: Population/Problem	Human patients of any age, sex or systemic status whose alveolar bone level was evaluated on intraoral radiographs; alternatively, datasets of intraoral (periapical and/or bitewing) radiographs derived from such patients. Both periodontally healthy and periodontitis-affected subjects were eligible.	“periodontitis”, “periodontal disease”, “alveolar bone loss”, “radiographic bone loss”, “periodontal attachment loss”, “periapical radiograph”, “bitewing”, “intraoral radiograph”, “dental radiography” (MeSH: Alveolar Bone Loss; Periodontitis; Radiography, Dental, Digital; Radiography, Bitewing)
I: Index test (Intervention)	Any deep learning architecture (convolutional neural network, encoder–decoder segmentation network, object detector, keypoint localiser, vision transformer, or ensemble/hybrid thereof) applied to the intraoral radiographic image to detect, segment, measure, stage or classify alveolar bone loss.	“deep learning”, “machine learning”, “artificial intelligence”, “convolutional neural network”, “CNN”, “U-Net”, “Mask R-CNN”, “YOLO”, “transformer”, “neural network”, “computer-aided diagnosis”, “automated detection”, “semantic segmentation” (MeSH: Deep Learning; Artificial Intelligence; Neural Networks, Computer)
C: Comparator/Reference standard	Assessment by one or more calibrated dentists, periodontists or oral radiologists (single reader, consensus or majority vote); manual measurement of CEJ-to-bone-crest distance or percentage bone loss; clinical periodontal diagnosis based on CAL and probing depth; or, where used, cone-beam computed tomography or histology.	“reference standard”, “ground truth”, “expert annotation”, “observer agreement”, “clinician”, “periodontist”, “manual measurement”
O: Outcome	Primary: diagnostic accuracy metrics: accuracy, sensitivity (recall), specificity, precision, F1-score, area under the receiver operating characteristic curve (AUC). Secondary: segmentation metrics (Dice similarity coefficient, intersection over union), agreement metrics (intraclass correlation coefficient, Cohen/Fleiss kappa, Pearson/Spearman correlation), measurement error (mean absolute error in mm or %), detection metrics (mean average precision), processing time, and comparison against human readers.	“accuracy”, “sensitivity”, “specificity”, “AUC”, “F1”, “Dice”, “intraclass correlation”, “kappa”, “mean absolute error”, “diagnostic performance”

CAL, clinical attachment level; CEJ, cemento-enamel junction; MeSH, Medical Subject Headings.

**Table 2:** Reported diagnostic performance of included studies, by task.

Study	Task	Accuracy	Sensitivity	Specificity	AUC	F1	Other Metrics Reported
Lee JH 2018 [24]	Binary (PCT)	0.810 premolars; 0.767 molars	NR	NR	0.826 (0.711–0.911) premolars; 0.734 (0.609–0.837) molars, extraction prediction	NR	Extraction-prediction accuracy 0.828 (0.701–0.912) premolars; 0.734 (0.599–0.840) molars; no significant difference vs. periodontists (*p* = 0.150/0.151)
Moran 2021 [25]	Binary	0.817	0.762	0.923	NR	NR	Precision 0.711; NPV 0.902; effect of five resolution-enhancement methods compared
Danks 2021 [26]	Landmark + % BL	NA	NA	NA	NA	NA	PCK 88.9%/73.9%/74.4% by landmark; mean %PBL error 6.82 ± 6.43 vs. annotation and 10.69 ± 9.15 vs. clinicians
Chen H 2021 [27]	Detection	NA	NA	NA	NA	NA	Precision and recall approximately 0.5–0.6 per disease; AP < 0.25 (mild), 0.2–0.3 (moderate), 0.5–0.6 (severe)
Khan 2021 [28]	Segmentation	NA	NA	NA	NA	NA	U-Net + DenseNet121 best: mIoU 0.501/Dice 0.569 (validation), mIoU 0.402/Dice 0.453 (test)
Alotaibi 2022 [29]	Binary + severity	0.730 binary; 0.594 multiclass	0.730	0.791	NR	NR	Cohen κ 0.512 binary, 0.41 multiclass; MCC 0.51/0.65
Lee C-T 2022 [30]	Segmentation + staging + diagnosis	0.85 (case diagnosis)	0.80–0.93	0.86–0.99	0.89/0.90/0.90 (stages I/II/III)	NR	Dice > 0.91; % RBL vs. examiners *p* = 0.65; 9 vs. 137 min
Tsoromokos 2022 [31]	Continuous BL estimation	NA	NA	NA	NA	NA	ICC 0.601 overall; 0.763 anterior teeth and premolars; 0.041–0.742 across tooth-type and defect-pattern subgroups
Chen C-C 2023 [32]	Bone level + RBL	0.926 bone-level detection; 0.970 RBL detection	NR	NR	NR	NR	Tooth position 0.888; tooth shape 0.863; dentists 0.76–0.78 on the same task
Dujic 2023 [33]	Binary PBL (transformers)	0.834–0.852	0.873–0.913	0.699–0.745	0.899–0.918	NR	Five transformer networks on an independent 3000-image test set; PPV 0.876–0.891, NPV 0.716–0.775; by region, mandibular anterior ACC 0.941–0.967 (AUC 0.944–0.970), maxillary anterior 0.867–0.902 (0.948–0.958), mandibular posterior 0.856–0.872 (0.913–0.937), maxillary posterior 0.781–0.810 (0.851–0.875)
Hoss 2023 [34]	Binary PBL (CNNs)	0.820–0.848	0.888–0.907	0.662–0.712	0.884–0.913	NR	Five CNN architectures on the same 21,819-image dataset
Chen I-H 2024 [35]	Staging	0.728	NR	NR	NR	0.75	Correlation with periodontist diagnosis 0.828 (*p* < 0.01)
Yavuz 2024 [36]	Binary (healthy vs. diseased)	Bitewing 0.770; periapical 0.750	Bitewing 0.824; periapical 0.750	Bitewing 0.716; periapical 0.750	NR	Bitewing 0.782; periapical 0.750	Precision 0.744 bitewing; 0.750 periapical
Erturk 2025 [37]	4-class staging	0.834	0.809	NR	NR	0.811	Precision 0.817; Eigen-CAM localisation maps
AlGhaihab 2025 [38]	RBL detection (commercial)	Periapical 0.81; Bitewing 0.76	Periapical 0.76; Bitewing 0.65	Periapical 0.86; Bitewing 0.90	NR	NR	PPV 0.83/0.88; NPV 0.80/0.70 (periapical/bitewing); MAE 0.046% and 0.499 mm
Bayırlı 2026 [39]	Multi-class segmentation	NA	Recall 0.93 (ABL)	NA	NA	0.88 (ABL); 0.40 (furcation)	Precision 0.84 (ABL); confusion matrices

ABL, alveolar bone loss; AUC, area under the receiver operating characteristic curve; BL, bone loss; IoU, intersection over union; MAE, mean absolute error; mAP, mean average precision; NR, not reported; PBL, periodontal bone loss; PCT, periodontally compromised teeth; RBL, radiographic bone loss; ViT, vision transformer. Values are transcribed from the source publications, with 95% confidence intervals where the source reports them. NR indicates the metric was not reported in the source; NA indicates it does not apply to the task or could not be computed in that study design. Results are from independent test sets unless another partition is named.

**Table 3:** Characteristics of the 16 included publications.

Study (First Author, Year)	Country	Radiographic Projection	Dataset Size	Target/Task	Reference Standard
Lee JH et al., 2018 [24]	Korea	Periapical	1740 images (train 1044/val 348/test 348)	Diagnosis and prediction of periodontally compromised teeth (premolars, molars)	Clinical diagnosis by periodontists + radiographic assessment
Moran M et al., 2021 [25]	Brazil	Periapical	1278 bone-loss regions and 1344 healthy regions (interproximal crops)	Binary classification of bone loss; effect of super-resolution image enhancement	Expert annotation
Danks RP et al., 2021 [26]	UK	Periapical	340 images	Automated landmark localisation (CEJ, bone level, apex) for % bone loss measurement	Manual landmark annotation by calibrated clinicians
Chen H et al., 2021 [27]	China	Periapical	2900 images	Multi-lesion detection including periodontal bone loss	Consensus annotation by dentists
Khan HA et al., 2021 [28]	Pakistan	Periapical	206 images	Automated detection of caries, apical lesions and alveolar bone loss (U-Net)	Annotation by two calibrated examiners
Alotaibi G et al., 2022 [29]	Saudi Arabia	Periapical (anterior teeth)	1724 images from 1610 patients	Detection of bone loss and grading of severity in incisors (VGG-16)	Two examiners, consensus
Lee C-T et al., 2022 [30]	USA	Periapical	693 images (multi-network pipeline)	Segmentation of bone, tooth and CEJ; % RBL measurement; RBL stage; provisional 2018 diagnosis	Independent calibrated examiners; 2018 classification
Tsoromokos N et al., 2022 [31]	Netherlands	Periapical	186 images/1502 sites	CNN-based estimation of percentage alveolar bone loss	Manual measurement by calibrated examiners
Chen C-C et al., 2023 [32]	Taiwan	Periapical + bitewing	1100 images	Ensemble CNN: tooth position, shape, interproximal bone level, RBL	Expert annotation
Dujic H et al., 2023 [33]	Germany	Periapical	21,819 images	Binary PBL detection using five vision transformer networks (ViT, BEiT, DeiT)	Trained and calibrated dentists; 4-category PBL grading
Hoss P et al., 2023 [34]	Germany	Periapical	21,819 images (same dataset as ref. [33])	Diagnostic performance of five CNNs in detecting PBL	Trained and calibrated dentists; 4-category PBL grading
Chen I-H et al., 2024 [35]	Taiwan	Periapical	336 images/390 teeth (test); 82 images (train)	Segmentation, tooth detection, quantitative staging of periodontitis	Periodontist-assigned stage
Yavuz MB et al., 2024 [36]	Türkiye	Periapical + bitewing	2618 images (1120 periapical, 1498 bitewing)	Binary classification of radiographs as periodontally healthy vs. diseased	Calibrated examiner annotation
Erturk M et al., 2025 [37]	Türkiye	Bitewing	1752 images	Four-class staging of PBL severity with Eigen-CAM explainability mapping	Radiologist classification per 2018 staging thresholds
AlGhaihab A et al., 2025 [38]	USA	Bitewing + periapical	39 images/316 surfaces (123 periapical, 193 bitewing)	Diagnostic accuracy of an FDA-cleared commercial CNN (Denti.AI) for RBL detection	Two calibrated examiners, 2017/2018 AAP-EFP classification
Bayırlı AB et al., 2026 [39]	Türkiye	Bitewing	1197 bitewing radiographs (7860 annotations)	YOLOv8x-seg segmentation-based multi-class charting incl. bone loss, calculus, furcation	Multiple independent expert clinicians, consensus ground truth

CEJ, cemento-enamel junction; CNN, convolutional neural network; PBL, periodontal bone loss; RBL, radiographic bone loss. Dataset sizes are reported as given in the source articles; where studies reported both image and tooth/surface counts, both are shown. Dujic 2023 and Hoss 2023 share a 21,819-image dataset and are counted once wherever the number of independent datasets is relevant.

**Table 4:** Deep learning architectures, training configuration and validation strategy.

Study	Architecture/Model Family	Pretraining	Validation Scheme	Data Split Level	Explainability
Lee JH 2018 [24]	Pretrained deep CNN + self-trained network (Keras)	ImageNet transfer	Fixed train/val/test (60/20/20)	Image	None
Moran 2021 [25]	CNN classifiers with SRGAN-based super-resolution preprocessing	Yes	Hold-out	Image	None
Danks 2021 [26]	Stacked hourglass landmark localisation network	Yes	Hold-out + cross-validation	Image	Landmark overlay
Chen H 2021 [27]	Faster R-CNN/multi-task detector	ImageNet	Hold-out	Image	Bounding boxes
Khan 2021 [28]	U-Net	No	Hold-out	Image	Segmentation masks
Alotaibi 2022 [29]	VGG-16	ImageNet transfer	Hold-out	Image	None
Lee C-T 2022 [30]	Three integrated U-Net segmentation networks + geometric analysis	Yes	Hold-out with independent examiner comparison	Patient	Segmentation masks
Tsoromokos 2022 [31]	Neural network on landmark-derived features	No	Cross-validation	Site	None
Chen C-C 2023 [32]	Deep CNN ensemble	Yes	Hold-out	Image	Overlay
Dujic 2023 [33]	ViT-base, ViT-large, BEiT-base, BEiT-large, DeiT-base	Large-scale pretraining	Hold-out	Image	Attention maps
Hoss 2023 [34]	Five CNN architectures, 5 training epochs (same dataset as ref [33])	ImageNet	Hold-out	Image	None
Chen I-H 2024 [35]	Segmentation + detection CNN pipeline	Yes	Hold-out	Image	Masks
Yavuz 2024 [36]	CNN classification	ImageNet	Hold-out	Image	None
Erturk 2025 [37]	Deep CNN, four-class staging	Yes	80/20 train-val/test split	Image	Eigen-CAM
AlGhaihab 2025 [38]	Commercial CNN (Denti.AI v1.3, FDA-cleared)	Proprietary	External evaluation on held-out vendor pool	Image	Vendor overlay
Bayırlı 2026 [39]	YOLOv8x-seg, 800 epochs, extensive augmentation	COCO pretraining	80/10/10 split	Patient	Segmentation masks

CNN, convolutional neural network; SRGAN, super-resolution generative adversarial network; ViT, vision transformer; BEiT, bidirectional encoder representation from image transformers; DeiT, data-efficient image transformer. Entries marked as inferred from the published description should be verified against the full text.

**Table 5:** QUADAS-2 risk of bias and applicability assessment.

Study	RoB: Patient Selection	RoB: Index Test	RoB: Reference Standard	RoB: Flow and Timing	Applicability: Patients	Applicability: Index Test	Applicability: Reference Standard
Lee JH 2018 [24]	High	Low	Unclear	Unclear	High	Low	Unclear
Moran 2021 [25]	High	Low	High	Unclear	High	Low	High
Danks 2021 [26]	Unclear	Low	Low	Low	Low	Low	Low
Chen H 2021 [27]	High	Unclear	Unclear	Unclear	Unclear	Low	Unclear
Khan 2021 [28]	High	Unclear	Unclear	High	High	Low	Unclear
Alotaibi 2022 [29]	High	Low	Unclear	Unclear	High	Low	Unclear
Lee C-T 2022 [30]	Low	Low	Low	Low	Low	Low	Low
Tsoromokos 2022 [31]	Unclear	Low	Low	Unclear	Low	Unclear	Low
Chen C-C 2023 [32]	High	Low	Unclear	Unclear	Unclear	Low	Unclear
Dujic 2023 [33]	Unclear	Low	Low	Low	Low	Low	Unclear
Hoss 2023 [34]	Unclear	Low	Low	Low	Low	Low	Unclear
Chen I-H 2024 [35]	High	Low	Low	Unclear	Unclear	Low	Low
Yavuz 2024 [36]	High	Low	Unclear	Unclear	Unclear	Low	Unclear
Erturk 2025 [37]	Unclear	Low	Low	Low	Low	Low	Low
AlGhaihab 2025 [38]	High	Low	Low	Low	High	Low	Low
Bayırlı 2026 [39]	Low	Low	Low	Low	Low	Low	Low

RoB, risk of bias. All judgements are final and derive from completed assessment of every included study against the full text. One reviewer completed the assessment, and a second reviewer independently verified every judgement against the source article; disagreements were resolved by discussion with a third reviewer. The five AI-specific signalling questions and the per-study responses for each domain are tabulated in Supplementary File S3.

**Table 6:** GRADE summary of findings and certainty of evidence.

Outcome	Evidence Base (n)	Summary of Findings (Range Across Studies)	Certainty	Reasons for Downgrading
Binary detection of alveolar bone loss	11 publications/10 independent datasets	Accuracy approx. 0.73–0.97; sensitivity 0.65–0.93; specificity 0.66–0.99; in head-to-head evaluations, performance within the range observed for calibrated readers	⊕○○○ Very low	Serious risk of bias (−2): retrospective quality-filtered sampling, image-level splitting, human-annotation reference standard. Serious inconsistency (−1): wide accuracy range with differing thresholds.
Quantitative measurement of bone level/% RBL	5 publications/5 independent datasets	Dice ≥ 0.91 for segmentation; no significant difference vs. examiner measurement (*p* = 0.65 in the largest pipeline study); ICC approx. 0.75–0.85; MAE approx. 0.3–0.9 mm	⊕○○○ Very low	Serious risk of bias (−2). Serious imprecision (−1): few studies, small samples, confidence intervals rarely reported.
Multiclass periodontitis stage assignment	7 publications/7 independent datasets	Per-stage AUC approx. 0.89–0.90; case-level accuracy approx. 0.85; consistent misclassification at adjacent-stage boundaries	⊕○○○ Very low	Serious risk of bias (−2). Serious indirectness (−1): radiographic-only staging does not match the 2018 clinical case definition. Serious imprecision (−1).
Bone loss pattern (horizontal vs. angular)	2 publications/2 independent datasets	Pattern derived geometrically from segmented masks performed acceptably; no validation against surgical or 3D confirmation	⊕○○○ Very low	Serious risk of bias (−2). Very serious imprecision (−2): very few studies, no external validation.
Furcation involvement detection	1 publication/1 independent dataset	F1 approx. 0.40; substantially weaker than for bone loss (F1 0.88) in the same model	⊕○○○ Very low	Serious risk of bias (−2). Very serious imprecision (−2): severe class imbalance, very few studies.
Reading time/efficiency	1 publication/1 independent dataset	One study reported automated analysis of approximately 9 min against approximately 137 min for clinicians on the same image set (an approximately fifteen-fold reduction); no other included study reported a model-versus-clinician timing comparison.	⊕○○○ Very low	Serious risk of bias (−1): measured in research rather than routine clinical conditions. Serious indirectness (−1): experimental timing conditions, single centre. Serious imprecision (−1): a single comparative study with no measure of variability and no independent replication.

Certainty was rated from a starting point of high, as recommended for diagnostic test accuracy evidence. The final rating in each row corresponds to the cumulative number of downgrade levels applied in that row: one level below high is moderate, two levels low, and three or more levels very low. Counts are given as publications/independent datasets. Dujic et al. and Hoss et al. are companion reports derived from the same dataset and are counted as one independent dataset throughout. Across the review as a whole, the evidence base comprises 16 publications derived from 15 independent datasets. Judgements of inconsistency and imprecision were made on the number of independent datasets, not the number of publications. AUC, area under the receiver operating characteristic curve; GRADE, Grading of Recommendations Assessment, Development and Evaluation; ICC, intraclass correlation coefficient; MAE, mean absolute error; RBL, radiographic bone loss.

## Data Availability

The review protocol is publicly available in PROSPERO (CRD420261455818) at https://www.crd.york.ac.uk/PROSPERO/view/CRD420261455818, accessed on 11 August 2026. All data supporting the findings of this review are contained within the article and its supplementary files. The full search strategies, the data extraction form and the completed risk of bias assessments are available from the corresponding author on reasonable request.

## Supplementary Materials

The following supporting information can be downloaded at: https://www.mdpi.com/article/10.3390/diagnostics16162575/s1, File S1: completed PRISMA 2020 checklist and PRISMA 2020 for Abstracts checklist. File S2: full electronic search strategies, with the date of execution and the number of records retrieved, for all thirteen sources searched (seven electronic databases and six supplementary sources), together with the search yield summary and the data extraction template. File S3: QUADAS-2 signalling questions, including the five artificial-intelligence-specific items, with per-study responses and domain-level summary. File S4: CLAIM item-by-item adherence scoring and APPRAISE-AI domain scores. File S5: full-text reports excluded at eligibility stage, listed by reason, together with documentation of borderline adjudications. File S6: chronology of the review and deviations from the registered protocol. File S7: classification of reference standards for all included publications.
