# Supplementary material for: Deep Learning for the Assessment of Alveolar Bone Loss on Intraoral Radiographs: A Systematic Review
Source: Diagnostics (Basel). 2026 Aug 14;16(16):2575. doi: 10.3390/diagnostics16162575 (PMC13512858; doi:10.3390/diagnostics16162575)
Supplement: Supplementary file 1 [file diagnostics-16-02575-s001.zip › S1-PRISMA-checklist.pdf]

Supplementary File S1. PRISMA 2020 Checklist

Manuscript: Deep Learning for the Assessment of Alveolar Bone Loss on Intraoral Radiographs: A Systematic Review.

Checklist source: Page MJ, McKenzie JE, Bossuyt PM, et al. The PRISMA 2020 statement: an updated guideline for reporting systematic reviews. BMJ. 2021;372:n71.

The final column identifies the section of the manuscript in which each item is reported. Section references are used in preference to page numbers so that the checklist remains accurate irrespective of pagination at typesetting.

Item	Section / topic	Checklist item	Location reported
**TITLE**			
1	Title	Identify the report as a systematic review.	Title page
**ABSTRACT**			
2	Abstract	See the PRISMA 2020 for Abstracts checklist (completed separately below).	Abstract
**INTRODUCTION**			
3	Rationale	Describe the rationale for the review in the context of existing knowledge.	Section 1, paras 1–5
4	Objectives	Provide an explicit statement of the objective(s) or question(s) the review addresses.	Section 1, final para; Section 2.2 (Table 1)
**METHODS**			
5	Eligibility criteria	Specify the inclusion and exclusion criteria for the review and how studies were grouped for the syntheses.	Section 2.3
6	Information sources	Specify all databases, registers, websites, organisations, reference lists and other sources searched or consulted, and the date when each source was last searched or consulted.	Section 2.4
7	Search strategy	Present the full search strategies for all databases, registers and websites, including any filters and limits used.	Section 2.4 (PubMed in text); Supplementary File S2
8	Selection process	Specify the methods used to decide whether a study met the inclusion criteria, including how many reviewers screened each record and each report, whether they worked independently, and, if applicable, details of automation tools used.	Section 2.5
9	Data collection process	Specify the methods used to collect data from reports, including how many reviewers collected data from each report, whether they worked independently, any processes for obtaining or confirming data from study investigators, and, if applicable, details of automation tools used.	Section 2.6
10a	Data items — outcomes	List and define all outcomes for which data were sought. Specify whether all results compatible with each outcome domain in each study were sought, and if not, the methods used to decide which results to collect.	Section 2.6; Table 1 (O element)
10b	Data items — other variables	List and define all other variables for which data were sought. Describe any assumptions made about any missing or unclear information.	Section 2.6; Supplementary File S2 (extraction template)
11	Study risk of bias assessment	Specify the methods used to assess risk of bias in the included studies, including details of the tool(s) used, how many reviewers assessed each study and whether they worked independently, and, if applicable, details of automation tools used.	Section 2.7; Supplementary File S3
12	Effect measures	Specify for each outcome the effect measure(s) used in the synthesis or presentation of results.	Section 2.8; Table 4
13a	Synthesis — eligibility	Describe the processes used to decide which studies were eligible for each synthesis.	Section 2.8
13b	Synthesis — data preparation	Describe any methods required to prepare the data for presentation or synthesis, such as handling of missing summary statistics or data conversions.	Section 2.6, Section 2.8
13c	Synthesis — tabulation	Describe any methods used to tabulate or visually display results of individual studies and syntheses.	Section 2.8; Tables 2–6
13d	Synthesis — methods	Describe any methods used to synthesise results and provide a rationale for the choice(s). If meta-analysis was performed, describe the model(s), method(s) to identify the presence and extent of statistical heterogeneity, and software package(s) used.	Section 2.8 (narrative synthesis per SWiM; meta-analysis not performed — rationale given)
13e	Synthesis — heterogeneity	Describe any methods used to explore possible causes of heterogeneity among study results.	Section 2.8; Section 4.4
13f	Synthesis — sensitivity analyses	Describe any sensitivity analyses conducted to assess robustness of the synthesised results.	Not applicable — no quantitative synthesis (Section 2.8)

Item	Section / topic	Checklist item	Location reported
14	Reporting bias assessment	Describe any methods used to assess risk of bias due to missing results in a synthesis (arising from reporting biases).	Section 2.9; Section 4.7
15	Certainty assessment	Describe any methods used to assess certainty (or confidence) in the body of evidence for an outcome.	Section 2.9; Table 6
**RESULTS**			
16a	Study selection — flow	Describe the results of the search and selection process, from the number of records identified in the search to the number of studies included in the review, ideally using a flow diagram.	Section 3.1; Figure 1
16b	Study selection — exclusions	Cite studies that might appear to meet the inclusion criteria, but which were excluded, and explain why they were excluded.	Section 3.1; Supplementary File S5
17	Study characteristics	Cite each included study and present its characteristics.	Section 3.2; Tables 2 and 3
18	Risk of bias in studies	Present assessments of risk of bias for each included study.	Section 3.5; Table 5; Supplementary Files S3 and S4
19	Results of individual studies	For all outcomes, present, for each study, summary statistics for each group (where appropriate) and an effect estimate and its precision (e.g. confidence/credible interval), ideally using structured tables or plots.	Section 3.4; Table 4
20a	Results of syntheses — characteristics	For each synthesis, briefly summarise the characteristics and risk of bias among contributing studies.	Sections 3.4.1–3.4.5
20b	Results of syntheses — statistical	Present results of all statistical syntheses conducted. If meta-analysis was done, present for each the summary estimate and its precision and measures of statistical heterogeneity.	Not applicable — narrative synthesis with ranges (Section 3.4; Table 6)
20c	Results of syntheses — heterogeneity	Present results of all investigations of possible causes of heterogeneity among study results.	Sections 3.4, 4.4
20d	Results of syntheses — sensitivity	Present results of all sensitivity analyses conducted to assess the robustness of the synthesised results.	Not applicable
21	Reporting biases	Present assessments of risk of bias due to missing results (arising from reporting biases) for each synthesis assessed.	Section 4.7
22	Certainty of evidence	Present assessments of certainty (or confidence) in the body of evidence for each outcome assessed.	Section 3.6; Table 6
**DISCUSSION**			
23a	General interpretation	Provide a general interpretation of the results in the context of other evidence.	Sections 4.1, 4.6
23b	Limitations of evidence	Discuss any limitations of the evidence included in the review.	Sections 4.3, 4.4, 4.5
23c	Limitations of review processes	Discuss any limitations of the review processes used.	Section 4.7
23d	Implications	Discuss implications of the results for practice, policy and future research.	Sections 4.8, 4.9; Section 5
**OTHER INFORMATION**			
24a	Registration	Provide registration information for the review, including register name and registration number, or state that the review was not registered.	Section 2.1 (PROSPERO [CRD420261455818])
24b	Protocol access	Indicate where the review protocol can be accessed, or state that a protocol was not prepared.	Section 2.1
24c	Amendments	Describe and explain any amendments to information provided at registration or in the protocol.	Section 2.10
25	Support	Describe sources of financial or non-financial support for the review, and the role of the funders or sponsors in the review.	Declarations — Funding
26	Competing interests	Declare any competing interests of review authors.	Declarations — Conflicts of interest
27	Availability of data, code and other materials	Report which of the following are publicly available and where they can be found: template data collection forms; data extracted from included studies; data used for all analyses; analytic code; any other materials used in the review.	Declarations — Data availability; Supplementary Files S1–S5

PRISMA 2020 for Abstracts Checklist

Item	Topic	Checklist item	Reported
1	Title	Identify the report as a systematic review.	Yes — title
2	Objectives	Provide an explicit statement of the main objective(s) or question(s) the review addresses.	Yes — Objectives
3	Eligibility criteria	Specify the inclusion and exclusion criteria for the review.	Yes — Methods
4	Information sources	Specify the information sources used to identify studies and the date when each was last searched.	Yes — Methods
5	Risk of bias	Specify the methods used to assess risk of bias in the included studies.	Yes — Methods (QUADAS-2, CLAIM, APPRAISE-AI)
6	Synthesis of results	Specify the methods used to present and synthesise results.	Yes — Methods (narrative synthesis; rationale given)
7	Included studies	Give the total number of included studies and participants and summarise relevant characteristics.	Yes — Results
8	Synthesis of results	Present results for main outcomes, preferably indicating the number of included studies and participants for each. If meta-analysis was done, report the summary estimate and confidence/credible interval.	Yes — Results (ranges, not pooled estimates)
9	Limitations of evidence	Provide a brief summary of the limitations of the evidence included in the review.	Yes — Results and Conclusions
10	Interpretation	Provide a general interpretation of the results and important implications.	Yes — Conclusions
11	Funding	Specify the primary source of funding for the review.	To be inserted
12	Registration	Provide the register name and registration number.	To be inserted (PROSPERO)
