# Supplementary material for: Deep Learning for the Assessment of Alveolar Bone Loss on Intraoral Radiographs: A Systematic Review
Source: Diagnostics (Basel). 2026 Aug 14;16(16):2575. doi: 10.3390/diagnostics16162575 (PMC13512858; doi:10.3390/diagnostics16162575)
Supplement: Supplementary file 1 [file diagnostics-16-02575-s001.zip › S2-search-strategies.pdf]

Supplementary File S2. Full Electronic Search Strategies and Data Extraction Template

Manuscript: Deep Learning for the Assessment of Alveolar Bone Loss on Intraoral Radiographs: A Systematic Review.

Searches were designed by the review team in consultation with a health sciences information specialist, combining three concept blocks — (A) deep learning / artificial intelligence, (B) alveolar bone loss / periodontitis, and (C) intraoral radiography — with the Boolean operator AND. Terms within each block were combined with OR. Controlled vocabulary (MeSH, Emtree) was exploded and combined with free-text terms searched in title, abstract and keyword fields. Date limits: 1 January 2015 to 12 June 2026. Language limit: English.

The exact date each source was last searched and the number of records retrieved are given in the summary table at the end of this file. These figures reconcile with Figure 1 of the main manuscript.

S2.1 MEDLINE via PubMed

```
((("deep learning"[MeSH Terms] OR "deep learning"[tiab] OR "machine learning"[MeSH Terms] OR
"artificial intelligence"[MeSH Terms] OR "artificial intelligence"[tiab] OR "neural networks,
computer"[MeSH Terms] OR "convolutional neural network*"[tiab] OR CNN[tiab] OR "U-Net"[tiab] OR
"Mask R-CNN"[tiab] OR "YOLO"[tiab] OR "vision transformer*"[tiab] OR "computer-aided
detection"[tiab] OR "computer-aided diagnos*"[tiab] OR "automated detection"[tiab] OR "semantic
segmentation"[tiab])
AND
("alveolar bone loss"[MeSH Terms] OR "alveolar bone loss"[tiab] OR "periodontal bone loss"[tiab]
OR "radiographic bone loss"[tiab] OR "bone level"[tiab] OR "crestal bone"[tiab] OR
"periodontitis"[MeSH Terms] OR "periodontitis"[tiab] OR "periodontal disease*"[tiab])
AND
("radiography, dental"[MeSH Terms] OR "radiography, bitewing"[MeSH Terms] OR "periapical
radiograph*"[tiab] OR "intraoral radiograph*"[tiab] OR "intra-oral radiograph*"[tiab] OR
"bitewing*"[tiab] OR "bite-wing*"[tiab] OR "dental radiograph*"[tiab] OR "dental X-ray*"[tiab])))
NOT ("review"[Publication Type] OR "case reports"[Publication Type])

Filters: English; 2015/01/01:2026/12/31[dp]
```

S2.2 Scopus

```
TITLE-ABS-KEY(("deep learning" OR "machine learning" OR "artificial intelligence" OR
"convolutional neural network*" OR "neural network*" OR CNN OR "U-Net" OR YOLO OR
"vision transformer*" OR "computer-aided diagnos*" OR "semantic segmentation")
AND ("alveolar bone loss" OR "periodontal bone loss" OR "radiographic bone loss" OR
"bone level" OR periodontitis OR "periodontal disease*")
AND ("periapical radiograph*" OR "intraoral radiograph*" OR "intra-oral radiograph*" OR
bitewing OR "bite-wing" OR "dental radiograph*"))
AND PUBYEAR > 2014
AND (LIMIT-TO(DOCTYPE,"ar") OR LIMIT-TO(DOCTYPE,"cp"))
AND (LIMIT-TO(LANGUAGE,"English"))
```

S2.3 Web of Science Core Collection

```
TS=(("deep learning" OR "machine learning" OR "artificial intelligence" OR
"convolutional neural network*" OR CNN OR "U-Net" OR YOLO OR "vision transformer*")
AND ("alveolar bone loss" OR "periodontal bone loss" OR "radiographic bone loss" OR
periodontitis)
AND ("periapical radiograph*" OR "intraoral radiograph*" OR bitewing OR "bite-wing" OR
"dental radiograph*"))
AND PY=(2015-2026)
Refined by: Document Types = (Article OR Proceedings Paper); Languages = (English)
```

S2.4 Embase (Ovid interface)

```
1  exp deep learning/
2  exp artificial intelligence/
3  exp machine learning/
4  (deep adj2 learning).ti,ab,kw.
5  (convolutional adj2 neural adj2 network*).ti,ab,kw.
6  ("U-Net" or "Mask R-CNN" or YOLO or "vision transformer*" or CNN).ti,ab,kw.
7  1 or 2 or 3 or 4 or 5 or 6
```

```
8  exp alveolar bone loss/
9  exp periodontitis/
10 (alveolar adj2 bone adj2 loss).ti,ab,kw.
11 (periodontal adj2 bone adj2 loss).ti,ab,kw.
12 (radiographic adj2 bone adj2 loss).ti,ab,kw.
13 8 or 9 or 10 or 11 or 12
14 exp bitewing radiography/
15 exp dental radiography/
16 (periapical adj2 radiograph*).ti,ab,kw.
17 (intraoral adj2 radiograph*).ti,ab,kw.
18 (bitewing* or bite-wing*).ti,ab,kw.
19 14 or 15 or 16 or 17 or 18
20 7 and 13 and 19
21 limit 20 to (english language and yr="2015 -Current")
22 limit 21 to (article or "conference paper")
```

S2.5 IEEE Xplore

```
(("All Metadata":"deep learning" OR "All Metadata":"convolutional neural network" OR
"All Metadata":"vision transformer" OR "All Metadata":"semantic segmentation")
AND ("All Metadata":"periodontal" OR "All Metadata":"alveolar bone loss" OR
"All Metadata":"periodontitis")
AND ("All Metadata":"dental radiograph" OR "All Metadata":"periapical" OR
"All Metadata":"bitewing"))

Filters: 2015–2026; Conferences, Journals, Early Access
```

S2.6 ACM Digital Library

```
[[All: "deep learning"] OR [All: "convolutional neural network"] OR [All: "transformer"]]
AND [[All: "periodontal"] OR [All: "alveolar bone loss"]]
AND [[All: "dental radiograph"] OR [All: "periapical"] OR [All: "bitewing"]]
AND [E-Publication Date: (01/01/2015 TO 12/31/2026)]
```

S2.7 Cochrane Library (CENTRAL)

```
#1  MeSH descriptor: [Alveolar Bone Loss] explode all trees
#2  MeSH descriptor: [Periodontitis] explode all trees
#3  MeSH descriptor: [Deep Learning] explode all trees
#4  MeSH descriptor: [Artificial Intelligence] explode all trees
#5  ("deep learning" OR "convolutional neural network" OR "artificial intelligence"):ti,ab,kw
#6  ("periapical radiograph*" OR "bitewing" OR "intraoral radiograph*"):ti,ab,kw
#7  (#1 OR #2) AND (#3 OR #4 OR #5) AND #6

Publication year from 2015 to 2026
```

S2.8 Google Scholar (supplementary)

```
allintitle: (deep learning OR CNN OR "artificial intelligence") (periodontal OR alveolar)
"bone loss" (radiograph OR radiographic)

First 200 results, sorted by relevance; screened for eligibility. Custom range 2015–2026.
```

S2.9 Preprint servers

```
arXiv:    all:("periodontal bone loss" OR "alveolar bone loss") AND all:("deep learning"
          OR "neural network")   [cs.CV, eess.IV categories]
medRxiv:  "periodontal bone loss" deep learning radiograph  (title/abstract search)
```

S2.10 Trial registries and grey literature

```
ClinicalTrials.gov:  Condition = periodontitis; Other terms = artificial intelligence OR
                     deep learning
ISRCTN:              periodontal AND (artificial intelligence OR deep learning)
OpenGrey / ProQuest Dissertations & Theses:  "alveolar bone loss" AND "deep learning"
```

S2.11 Hand-searching

```
Last five volumes of: Journal of Clinical Periodontology; Journal of Periodontology;
Journal of Dentistry; Dentomaxillofacial Radiology; Oral Radiology; Journal of Dental
```

Sciences; BMC Oral Health; Diagnostics (MDPI).

Plus backward citation chasing of all included studies and of relevant reviews, and forward citation chasing via Scopus and Google Scholar "cited by".

S2.12 Search yield summary

Source	Date last searched	Records retrieved	Records after de-duplication
MEDLINE / PubMed	12 June 2026	341	—
Scopus	12 June 2026	402	—
Web of Science	12 June 2026	356	—
Embase	12 June 2026	208	—
IEEE Xplore	12 June 2026	118	—
ACM Digital Library	12 June 2026	34	—
Cochrane Library	12 June 2026	23	—
Other methods (Google Scholar first 200, preprint servers, registries and grey literature, citation chasing and hand-searching)	12 June 2026	61	—
TOTAL	12 June 2026	1,543	946

S2.13 Data extraction template

Field	Description / permitted values
Study ID	First author surname + year of publication
Full citation	Vancouver format
Country / number of centres	Free text; single-centre vs multicentre
Design	Retrospective / prospective; development only / development + internal validation / external validation
Funding and commercial involvement	Free text; note any author affiliation with a vendor
Radiographic projection	Periapical / bitewing / mixed intraoral
Sensor type	PSP plate / CMOS / CCD / digitised film / not reported
Image resolution and preprocessing	Pixel dimensions; resizing, normalisation, contrast handling
n images / n teeth / n surfaces / n patients	Integers; state which unit was the unit of analysis
Image inclusion and exclusion criteria	Note any exclusion for image quality, restorations, tooth type
Train / validation / test split	Ratio and absolute numbers
Split level	Patient-level / image-level / not stated — MANDATORY FIELD
Cross-validation	None / k-fold (state k) / repeated group shuffling
Data augmentation	Type and extent
Architecture	Family + specific network + backbone
Pretraining	None / ImageNet / COCO / domain-specific
Loss function, optimiser, learning rate, epochs, batch size	As reported
Hardware and framework	GPU; TensorFlow / PyTorch / Keras and version
Reference standard	Number, discipline and years of experience of annotators
Annotator calibration	Method and reported reliability
Consensus method	Single reader / majority vote / consensus discussion / senior adjudication
Clinical data informing ground truth	Yes (specify: CAL, PPD, diagnosis) / No
Inter-annotator agreement	Cohen or Fleiss kappa / ICC / not reported
Task	Binary detection / multiclass staging / continuous measurement / segmentation / pattern / furcation
Threshold definition	How "bone loss" was dichotomised or categorised
Disease prevalence in test set	Percentage — MANDATORY FIELD for interpreting accuracy
Performance metrics	Accuracy, sensitivity, specificity, PPV, NPV, F1, AUC, Dice, IoU, ICC, MAE, mAP; record 95% CI where reported
Performance by subgroup	By tooth type, region, stage, projection where reported
Human reader comparison	Yes/No; number and experience of readers; statistical test; result
Processing time	Model vs human, with units
Explainability method	None / Grad-CAM / Eigen-CAM / attention maps / segmentation overlay
External validation	Yes/No; source, sensor and population of external data
Code availability	Public (URL) / on request / not available
Data availability	Public (URL) / on request / not available
Ethical approval	Yes (reference number) / No / not stated
QUADAS-2 judgements	Four risk of bias domains + three applicability domains
CLAIM adherence	n items reported / n applicable (%)
APPRAISE-AI score	0–100 and quality band
Reviewer initials and extraction date	Free text
Discrepancies and resolution	Free text
