# Supplementary material for: Deep Learning for the Assessment of Alveolar Bone Loss on Intraoral Radiographs: A Systematic Review
Source: Diagnostics (Basel). 2026 Aug 14;16(16):2575. doi: 10.3390/diagnostics16162575 (PMC13512858; doi:10.3390/diagnostics16162575)
Supplement: Supplementary file 1 [file diagnostics-16-02575-s001.zip › S3-QUADAS2.pdf]

Supplementary File S3

QUADAS-2 risk of bias and applicability, with artificial-intelligence-specific signalling questions

Deep Learning for the Assessment of Alveolar Bone Loss on Intraoral Radiographs: A Systematic Review. Manuscript diagnostics-4481953.

Risk of bias was assessed independently in duplicate for all 16 included publications using QUADAS-2, tailored to artificial intelligence diagnostic accuracy studies by the addition of five signalling questions. Each of the four risk-of-bias domains was rated low, high or unclear; applicability concerns were rated for the first three domains. Disagreements were resolved by a third reviewer.

Table S3.1. Domain-level QUADAS-2 judgements for the 16 included publications.

Study	RoB: Patient selection	RoB: Index test	RoB: Reference standard	RoB: Flow and timing	App: Patient selection	App: Index test	App: Reference standard
Lee JH 2018	High	High	Low	Low	High	Low	Low
Moran 2021	High	High	Unclear	High	High	Low	Low
Danks 2021	High	High	Low	Low	High	Low	Low
Chen H 2021	High	High	High	High	High	Low	High
Khan 2021	High	Low	Low	Low	High	Low	Low
Alotaibi 2022	High	High	Low	Low	High	High	Low
Lee C-T 2022	High	High	Low	Low	High	Low	Low
Tsoromokos 2022	High	High	Low	Low	High	Low	Low
Chen C-C 2023	High	High	Unclear	High	High	Low	Low
Dujic 2023	High	High	Low	High	High	Low	Low
Hoss 2023	High	High	Low	High	High	Low	Low
Chen I-H 2024	High	High	Low	Unclear	High	Low	Low
Yavuz 2024	High	High	Low	Unclear	High	Low	Low
Erturk 2025	High	High	Low	Low	High	Low	Low
AlGhaihab 2025	High	Low	Low	Low	High	Low	Low
Bayirli 2026	High	High	Low	Low	High	Low	Low

Table S3.2. Artificial-intelligence-specific signalling questions, per study.

Study	(i) Partitions disjoint at patient level	(ii) External or multicentre test set	(iii) Annotators blinded to model output and clinical data	(iv) Consecutive or random image sampling	(v) Sample size justified
Lee JH 2018	Internal retrospective split	No	Not reported	Quality-filtered or not stated	No
Moran 2021	Single-source internal evaluation	No	Not reported	Quality-filtered or not stated	No
Danks 2021	Internal only	No	Not reported	Quality-filtered or not stated	No
Chen H 2021	Fivefold image-level CV; no held-out set	No	Not reported	Quality-filtered or not stated	No
Khan 2021	External institutions	Yes	Not reported	Quality-filtered or not stated	No
Alotaibi 2022	Internal	No	Not reported	Quality-filtered or not stated	No
Lee C-T 2022	Internal	No	Not reported	Quality-filtered or not stated	No
Tsoromokos 2022	Internal	No	Not reported	Quality-filtered or not stated	No
Chen C-C 2023	Unclear patient-level independence	No	Not reported	Quality-filtered or not stated	No
Dujic 2023	No demonstrated patient-level separation	No	Not reported	Quality-filtered or not stated	No
Hoss 2023	No demonstrated patient-level separation	No	Not reported	Quality-filtered or not stated	No
Chen I-H 2024	Unclear/high flow concern	No	Not reported	Quality-filtered or not stated	No
Yavuz 2024	Unclear/high flow concern	No	Not reported	Quality-filtered or not stated	No
Erturk 2025	Internal	No	Not reported	Quality-filtered or not stated	No
AlGhaihab 2025	External clinical evaluation	Yes	Not reported	Quality-filtered or not stated	No
Bayirli 2026	Internal	No	Not reported	Quality-filtered or not stated	No

Notes. Question (ii) is taken from CLAIM item 32 (validation or testing on external data), scored as reported for two publications only. Question (iii) is answered "not reported" for every publication because no included report states whether annotators were blinded to model output or to clinical information. Question (iv) reflects that every included study applied quality-based image selection criteria or did not describe its sampling frame; this is the basis for the high

risk-of-bias rating in the patient-selection domain across all 16 studies. Question (v) is taken from CLAIM item 19 (intended sample size and how it was determined), scored as not reported in every included study.

Two structural findings follow from these questions. First, only a minority of studies demonstrate that data partitions are disjoint at the patient level; the remainder split at image level or do not state the level, so radiographs from one patient may appear in both training and test partitions and reported precision is correspondingly optimistic. Second, external validation on data from a different institution, sensor or population was performed in two studies only.
