# Supplementary material for: Deep Learning for the Assessment of Alveolar Bone Loss on Intraoral Radiographs: A Systematic Review
Source: Diagnostics (Basel). 2026 Aug 14;16(16):2575. doi: 10.3390/diagnostics16162575 (PMC13512858; doi:10.3390/diagnostics16162575)
Supplement: Supplementary file 1 [file diagnostics-16-02575-s001.zip › S4-CLAIM-APPRAISE-AI.pdf]

Supplementary File S4

CLAIM item-by-item adherence and APPRAISE-AI domain scores

Deep Learning for the Assessment of Alveolar Bone Loss on Intraoral Radiographs: A Systematic Review. Manuscript diagnostics-4481953.

Reporting completeness was assessed against the 42-item Checklist for Artificial Intelligence in Medical Imaging (CLAIM). Each item was scored as reported (R, 1.0 point), partially reported (PR, 0.5), not reported (NR, 0) or not applicable (NA, excluded from the denominator). Adherence is the points scored divided by the number of applicable items. Two reviewers scored independently and disagreements were resolved by a third reviewer.

Table S4.1. CLAIM item-by-item scoring for the 16 included publications.

#	CLAIM item	Lee JH 2018	Moran 2021	Danks 2021	Chen H 2021	Khan 2021	Alotaibi 2022	Lee C-T 2022	Tsorom. 2022	Chen C-C 2023	Dujic 2023	Hoss 2023	Chen I-H 2024	Yavuz 2024	Erturk 2025	AlGhaih. 2025	Bayirli 2026
1	Identification as a study of AI methodology, specifying the model category and imaging modality	R	R	R	R	R	R	R	R	R	R	R	R	R	R	R	R
2	Structured summary of study design, methods, results and conclusions	R	R	R	R	R	R	R	R	R	R	R	R	R	R	R	R
3	Scientific and clinical background, including intended use and clinical role of the model	R	R	R	R	R	R	R	R	R	R	R	R	R	R	R	R
4	Study objectives and hypotheses	R	R	R	R	R	R	R	R	R	R	R	R	R	R	R	R
5	Prospective or retrospective study	R	R	R	PR	PR	R	R	PR	PR	R	R	PR	R	R	R	R
6	Study goal: model creation, exploratory study, feasibility, non-inferiority trial	R	R	R	R	R	R	R	R	R	R	R	R	R	R	R	R
7	Data sources	R	R	R	R	R	R	R	R	R	R	R	R	R	R	R	R
8	Eligibility criteria: how, where and when eligible patients or images were identified	PR	PR	R	R	R	R	PR	R	PR	R	R	PR	R	R	R	R
9	Data pre-processing steps	PR	R	R	PR	R	PR	R	PR	R	R	R	R	R	R	PR	R
10	Selection of data subsets, if applicable	R	PR	R	R	R	R	R	R	R	R	R	R	R	R	PR	R
11	Definitions of data elements, with references to Common Data Elements	PR	PR	PR	R	PR	R	R	R	PR	R	R	R	PR	R	R	R
12	De-identification methods	PR	PR	R	R	R	PR	PR	R	R	R	R	R	R	PR	R	R
13	How missing data were handled	NR	NR	NR	NR	NR	NR	NR	NR	NR	NR	NR	NR	NR	NR	NR	NR
14	Definition of the reference standard, in sufficient detail to allow replication	R	R	R	R	R	R	R	R	R	R	R	R	R	R	R	R
15	Rationale for choosing the reference standard	PR	PR	PR	PR	PR	PR	R	PR	PR	R	R	PR	PR	R	R	R
16	Source of ground-truth annotations; qualifications and preparation of annotators	R	R	R	R	R	R	R	R	R	R	R	R	R	R	R	R
17	Annotation tools	PR	PR	R	PR	R	PR	R	R	R	R	R	R	R	R	PR	R
18	Measurement of inter- and intra-rater variability; how disagreements were resolved	PR	PR	R	NR	PR	PR	R	R	PR	R	R	R	R	PR	R	R
19	Intended sample size and how it was determined	NR	NR	NR	NR	NR	NR	NR	NR	NR	NR	NR	NR	NR	NR	NR	NR
20	How data were assigned to partitions; specify proportions	R	R	R	R	R	R	R	R	R	R	R	R	R	R	PR	R
21	Level at which partitions are disjoint (e.g. images, patients, institutions)	R	PR	R	NR	R	R	R	R	PR	NR	NR	PR	PR	R	R	R
22	Detailed description of model, including inputs, outputs, layers and parameters	PR	R	R	R	R	PR	R	PR	R	R	R	R	R	R	PR	R

#	CLAIM item	Lee JH 2018	Moran 2021	Danks 2021	Chen H 2021	Khan 2021	Alotaibi 2022	Lee C-T 2022	Tsorom. 2022	Chen C-C 2023	Dujic 2023	Hoss 2023	Chen I-H 2024	Yavuz 2024	Erturk 2025	AlGhaih. 2025	Bayirli 2026
23	Software libraries, frameworks and packages	PR	R	R	R	R	PR	R	PR	R	R	R	R	R	R	PR	R
24	Initialisation of model parameters	PR	R	R	R	PR	PR	R	PR	R	R	R	R	R	R	PR	R
25	Details of training approach, including data augmentation and hyperparameters	PR	R	R	R	R	PR	R	PR	R	R	R	R	R	R	PR	R
26	Method of selecting the final model	PR	R	R	R	R	PR	R	PR	R	R	R	R	R	R	PR	R
27	Ensembling techniques, if applicable	NA	NA	NA	NA	NA	NA	R	NA	R	NA	NA	NA	NA	R	NA	NA
28	Metrics of model performance	R	R	R	R	R	R	R	R	R	R	R	R	R	R	R	R
29	Statistical measures of significance and uncertainty (e.g. confidence intervals)	R	PR	PR	PR	PR	PR	PR	R	PR	PR	PR	R	R	PR	R	PR
30	Robustness or sensitivity analysis	PR	R	R	R	R	PR	R	PR	R	R	R	PR	PR	R	PR	R
31	Methods for explainability or interpretability	NR	NR	PR	NR	NR	NR	PR	NR	NR	NR	NR	PR	NR	R	NR	PR
32	Validation or testing on external data	NR	NR	NR	NR	R	NR	NR	NR	NR	NR	NR	NR	NR	NR	R	NR
33	Flow of participants or cases; use of a diagram to indicate inclusion and exclusion	PR	PR	PR	PR	R	PR	PR	PR	PR	R	R	PR	PR	R	R	R
34	Demographic and clinical characteristics of cases in each partition	R	NR	NR	NR	NR	R	PR	PR	NR	PR	PR	PR	PR	NR	R	NR
35	Performance metrics for optimal model on all data partitions	R	R	R	R	R	R	R	R	R	R	R	R	R	R	R	R
36	Estimates of diagnostic accuracy and their precision	R	PR	PR	PR	PR	PR	PR	R	PR	PR	PR	R	R	PR	R	PR
37	Failure analysis of incorrectly classified cases	PR	PR	R	PR	R	PR	R	PR	PR	PR	PR	PR	PR	PR	PR	R
38	Study limitations, including potential bias, statistical uncertainty and generalisability	R	R	R	R	R	R	R	R	R	R	R	R	R	R	R	R
39	Implications for practice, including intended use and clinical role	R	R	R	R	R	R	R	R	R	R	R	R	R	R	R	R
40	Registration number and name of registry	NR	NR	NR	NR	NR	NR	NR	NR	NR	NR	NR	NR	NR	NR	NR	NR
41	Where the full study protocol can be accessed	NR	NR	NR	NR	NR	NR	NR	NR	NR	NR	NR	NR	NR	NR	NR	NR
42	Sources of funding and other support; role of funders	R	R	R	R	R	R	R	R	R	R	R	R	R	R	R	R
	Adherence (% of applicable items)	67.1%	68.3%	78.0%	68.3%	76.8%	67.1%	79.8%	70.7%	71.4%	78.0%	78.0%	76.8%	76.8%	79.8%	74.4%	81.7%

Table S4.2. APPRAISE-AI domain scores, totals and quality bands.

Study	Clinical relevance (0-4)	Data quality (0-20)	Methodological conduct (0-20)	Robustness of results (0-20)	Reporting quality (0-16)	Reproducibility (0-20)	Total (0-100)	Band
Lee JH 2018	4	16	10	9	10	9	58	Intermediate
Moran 2021	4	12	11	10	9	11	57	Intermediate
Danks 2021	4	15	12	12	10	13	66	High
Chen H 2021	4	10	8	8	8	12	50	Intermediate
Khan 2021	4	18	14	12	10	13	71	High
Alotaibi 2022	4	15	10	8	9	9	55	Intermediate
Lee C-T 2022	4	17	13	12	10	14	70	High
Tsoromokos 2022	4	14	11	10	10	10	59	Intermediate
Chen C-C 2023	4	14	11	10	9	12	60	High
Dujic 2023	4	15	8	9	10	14	60	High
Hoss 2023	4	15	8	9	10	14	60	High
Chen I-H 2024	4	14	10	10	10	13	61	High

Study	Clinical relevance (0-4)	Data quality (0-20)	Methodological conduct (0-20)	Robustness of results (0-20)	Reporting quality (0-16)	Reproducibility (0-20)	Total (0-100)	Band
Yavuz 2024	4	15	10	10	10	12	61	High
Erturk 2025	4	15	13	14	10	14	70	High
AlGhaihab 2025	4	18	15	14	11	10	72	High
Bayirli 2026	4	16	12	13	10	14	69	High

Bands follow Kwong and colleagues: very low 0-19; low 20-39; intermediate 40-59; high 60-79; very high 80-100. CLAIM measures whether a study was adequately reported; APPRAISE-AI measures whether its methodology was sound. The two are not interchangeable, and neither substitutes for the QUADAS-2 risk-of-bias assessment in Supplementary File S3.
