# Supplementary material for: Deep Learning for the Assessment of Alveolar Bone Loss on Intraoral Radiographs: A Systematic Review
Source: Diagnostics (Basel). 2026 Aug 14;16(16):2575. doi: 10.3390/diagnostics16162575 (PMC13512858; doi:10.3390/diagnostics16162575)
Supplement: Supplementary file 1 [file diagnostics-16-02575-s001.zip › S5-excluded-studies.pdf]

# Supplementary File S5

## Full-text reports excluded, with the principal reason for each

*Deep Learning for the Assessment of Alveolar Bone Loss on Intraoral Radiographs: A Systematic Review. Manuscript diagnostics-4481953.*

Seventy-three full-text reports were assessed for eligibility; 16 were included and 57 were excluded. Each excluded report is listed once and assigned a single principal reason, determined by applying the predefined exclusion hierarchy strictly in order and taking the first applicable criterion.

Hierarchy applied: (1) panoramic, CBCT, multislice CT, intraoral photographs or other non-intraoral-radiographic input used exclusively; (2) bone loss not the target condition, with no separately reported bone-loss output; (3) classical machine learning or image processing only, with no deep learning component; (4) narrative review, systematic review, editorial, letter, commentary, conference abstract without full methods, case report or technical note without quantitative validation; (5) in vitro, phantom or animal study; (6) no extractable performance metric or no defined reference standard; (7) duplicate or companion report of an included dataset.

**Category totals: non-intraoral modality 29; bone loss not the target condition 14; review, editorial or conference abstract 9; no deep learning component 5. Total 57. These totals correspond to Section 3.1 and to Figure 1. Following a full re-audit of the excluded records at revision, no report was excluded solely for want of an extractable performance metric or as a duplicate publication of an included dataset, and those two categories are therefore empty.**

| No. | Full citation | Principal reason for exclusion |
|---|---|---|
| 1 | Krois J, Ekert T, Meinhold L, Golla T, Kharbot B, Wittemeier A, et al. Deep learning for the radiographic detection of periodontal bone loss. Sci Rep. 2019;9(1):8495. | Panoramic radiographs used exclusively (non-intraoral modality) |
| 2 | Kim J, Lee HS, Song IS, Jung KH. DeNTNet: deep neural transfer network for the detection of periodontal bone loss using panoramic dental radiographs. Sci Rep. 2019;9(1):17615. | Panoramic radiographs used exclusively (non-intraoral modality) |
| 3 | Ekert T, Krois J, Meinhold L, Elhennawy K, Emara R, Golla T, Schwendicke F. Deep learning for the radiographic detection of apical lesions. J Endod. 2019;45(7):917-22.e5. | Panoramic radiographs used exclusively (non-intraoral modality) |
| 4 | Thanathornwong B, Suebnukarn S. Automatic detection of periodontal compromised teeth in digital panoramic radiographs using faster regional convolutional neural networks. Imaging Sci Dent. 2020;50(2):169-74. | Panoramic radiographs used exclusively (non-intraoral modality) |
| 5 | Chang HJ, Lee SJ, Yong TH, Shin NY, Jang BG, Kim JE, et al. Deep learning hybrid method to automatically diagnose periodontal bone loss and stage periodontitis. Sci Rep. 2020;10(1):7531. | Panoramic radiographs used exclusively (non-intraoral modality) |
| 6 | Li H, Zhou J, Zhou Y, Chen Q, She Y, Gao F, et al. An interpretable computer-aided diagnosis method for periodontitis from panoramic radiographs. Front Physiol. 2021;12:655556. | Panoramic radiographs used exclusively (non-intraoral modality) |
| 7 | Kuwana R, Ariji Y, Fukuda M, Kise Y, Nozawa M, Kuwada C, et al. Performance of deep learning object detection technology in the detection and diagnosis of maxillary sinus lesions on panoramic radiographs. Dentomaxillofac Radiol. 2021;50(1):20200171. | Panoramic radiographs used exclusively (non-intraoral modality) |
| 8 | Ezhov M, Gusarev M, Golitsyna M, Yates JM, Kushnerev E, Tamimi D, et al. Clinically applicable artificial intelligence system for dental diagnosis with CBCT. Sci Rep. 2021;11(1):15006. | Panoramic radiographs used exclusively (non-intraoral modality) |
| 9 | Jiang L, Chen D, Cao Z, Wu F, Zhu H, Zhu F. A two-stage deep learning architecture for radiographic staging of periodontal bone loss. BMC Oral Health. 2022;22(1):106. | Panoramic radiographs used exclusively (non-intraoral modality) |

| No. | Full citation | Principal reason for exclusion |
|---|---|---|
| 10 | Shon HS, Kong V, Park JS, Jang W, Cha EJ, Kim SY, et al. Deep learning model for classifying periodontitis stages on dental panoramic radiography. Appl Sci. 2022;12(17):8500. | Panoramic radiographs used exclusively (non-intraoral modality) |
| 11 | Widyaningrum R, Candradewi I, Aji NRAS, Aulianisa R. Comparison of multi-label U-Net and Mask R-CNN for panoramic radiograph segmentation to detect periodontitis. Imaging Sci Dent. 2022;52(4):383-91. | Panoramic radiographs used exclusively (non-intraoral modality) |
| 12 | Sunnetci KM, Ulukaya S, Alkan A. Periodontal bone loss detection based on hybrid deep learning and machine learning models with a user-friendly application. Biomed Signal Process Control. 2022;77:103844. | Panoramic radiographs used exclusively (non-intraoral modality) |
| 13 | Kearney VP, Yansane AM, Brandon RG, Vaderhobli R, Lin GH, Hekmatian H, et al. A generative adversarial inpainting network to enhance prediction of periodontal clinical attachment level. J Dent. 2022;123:104211. | Panoramic radiographs used exclusively (non-intraoral modality) |
| 14 | Ryu J, Lee DM, Jung YH, Kwon O, Park S, Hwang J, et al. Automated detection of periodontal bone loss using deep learning and panoramic radiographs: a convolutional neural network approach. Appl Sci. 2023;13(9):5261. | Panoramic radiographs used exclusively (non-intraoral modality) |
| 15 | Kong Z, Ouyang H, Cao Y, Huang T, Ahn E, Zhang M, Liu H. Automated periodontitis bone loss diagnosis in panoramic radiographs using a bespoke two-stage detector. Comput Biol Med. 2023;152:106374. | Panoramic radiographs used exclusively (non-intraoral modality) |
| 16 | Uzun Saylan BC, Baydar O, Yesilova E, Kurt Bayrakdar S, Bilgir E, Bayrakdar IS, et al. Assessing the effectiveness of artificial intelligence models for detecting alveolar bone loss in periodontal disease: a panoramic radiograph study. Diagnostics (Basel). 2023;13(10):1800. | Panoramic radiographs used exclusively (non-intraoral modality) |
| 17 | Orhan K, Belgin CA, Manulis D, Golitsyna M, Bayrak S, Aksoy S, et al. Determining the reliability of diagnosis and treatment using artificial intelligence software with panoramic radiographs. Imaging Sci Dent. 2023;53(3):199-208. | Panoramic radiographs used exclusively (non-intraoral modality) |
| 18 | Yarkac FU, Tassoker M, Atay UT, Azman D. Evaluation of trabecular bone in individuals with periodontitis using fractal analysis: an observational cross-sectional study. J Clin Exp Dent. 2023;15(12):e1022-8. | Panoramic radiographs used exclusively (non-intraoral modality) |
| 19 | Kurt-Bayrakdar S, Bayrakdar IS, Yavuz MB, Sali N, Celik O, Kose O, et al. Detection of periodontal bone loss patterns and furcation defects from panoramic radiographs using deep learning algorithm: a retrospective study. BMC Oral Health. 2024;24(1):155. | Panoramic radiographs used exclusively (non-intraoral modality) |
| 20 | Beser B, Reis T, Berber MN, Topaloglu E, Gungor E, Coruh Kilic M, et al. YOLO-V5 based deep learning approach for tooth detection and segmentation on pediatric panoramic radiographs in mixed dentition. BMC Med Imaging. 2024;24(1):172. | Panoramic radiographs used exclusively (non-intraoral modality) |
| 21 | Xue T, Chen L, Sun Q. Deep learning method to automatically diagnose periodontal bone loss and periodontitis stage in dental panoramic radiograph. J Dent. 2024;150:105373. | Panoramic radiographs used exclusively (non-intraoral modality) |
| 22 | Kong V, Lee EY, Kim KA, Shon HS. Integrating super-resolution with deep learning for enhanced periodontal bone loss segmentation in panoramic radiographs. Bioengineering (Basel). 2024;11(11):1130. | Panoramic radiographs used exclusively (non-intraoral modality) |
| 23 | Xi R, Ali M, Zhou Y, Tizzano M. A reliable deep-learning-based method for alveolar bone quantification using a murine model of periodontitis and micro-computed tomography imaging. J Dent. 2024;146:105057. | Panoramic radiographs used exclusively (non-intraoral modality) |
| 24 | Ragab MG, Abdulkadir SJ, Qaid N, Gondal TM, Alqushaibi A, Qureshi R, et al. Periodontitis bone loss detection in panoramic radiographs using modified YOLOv7. PeerJ Comput Sci. 2025;11:e3102. | Panoramic radiographs used exclusively (non-intraoral modality) |
| 25 | Putra RH, Astuti ER, Wijaksana IKE, Fariza A, Sitalaksmi RM, Yoda N. Automated periodontal bone loss detection on panoramic radiographs using | Panoramic radiographs used exclusively (non-intraoral modality) |

| No. | Full citation | Principal reason for exclusion |
|---|---|---|
| | You Only Look Once v8 (YOLOv8): a retrospective AI approach. J Int Oral Health. 2025;17(3):203-11. | |
| 26 | Widyaningrum R, Astuti ER, Soetojo A, Faadiya AN, Nurrachman AS, Kinanggit ND, et al. Hybrid two-stage CNN for detection and staging of periodontitis on panoramic radiographs. J Oral Biol Craniofac Res. 2025;15(6):1392-9. | Panoramic radiographs used exclusively (non-intraoral modality) |
| 27 | Mohammed M, Fernandez-Medina T, Rajashekhar M, Baker S, Jennings E. Alveolar bone segmentation methods in assessing the effectiveness of periodontal defect regeneration through machine learning of CBCT data: a systematic review. Int J Biomed Imaging. 2025;2025:9065572. | Panoramic radiographs used exclusively (non-intraoral modality) |
| 28 | Le MH, Mai XH, Tumur-Ulzii B, Kim SH, Oh NS. Automated periodontitis diagnosis and staging using an end-to-end deep learning model on panoramic dental radiographs. Oral Radiol. 2026;42:801-9. | Panoramic radiographs used exclusively (non-intraoral modality) |
| 29 | Madani Z, Fakhar HB. Detection and classification of peri-implant marginal bone loss in cone-beam computed tomography using a deep learning approach. Clin Exp Dent Res. 2026;12(2):e70308. | Panoramic radiographs used exclusively (non-intraoral modality) |
| 30 | Cantu AG, Gehrung S, Krois J, Chaurasia A, Rossi JG, Gaudin R, et al. Detecting caries lesions of different radiographic extension on bitewings using deep learning. J Dent. 2020;100:103425. | Bone loss not the target condition |
| 31 | Karatas O, Cakir NN, Ozsariyildiz SS, Kis HC, Demirbuga S, Gurgan CA. A deep learning approach to dental restoration classification from bitewing and periapical radiographs. Quintessence Int. 2021;52(7):568-74. | Bone loss not the target condition |
| 32 | Lee S, Oh S, Jo J, Kang S, Shin Y, Park JW. Deep learning for early dental caries detection in bitewing radiographs. Sci Rep. 2021;11:16807. | Bone loss not the target condition |
| 33 | Li CW, Lin SY, Chou HS, Chen TY, Chen YA, Liu SY, et al. Detection of dental apical lesions using CNNs on periapical radiograph. Sensors (Basel). 2021;21(21):7049. | Bone loss not the target condition |
| 34 | Bayraktar Y, Ayan E. Diagnosis of interproximal caries lesions with deep convolutional neural network in digital bitewing radiographs. Clin Oral Investig. 2022;26(1):623-32. | Bone loss not the target condition |
| 35 | Kabir T, Lee CT, Chen L, Jiang X, Shams S. A comprehensive artificial intelligence framework for dental diagnosis and charting. BMC Oral Health. 2022;22(1):480. Reclassified at revision: evaluates tooth-number recognition and full-mouth-series arrangement; bone and cemento-enamel junction masks serve tooth localisation and no bone-loss diagnostic output is separately reported. | Bone loss not the target condition |
| 36 | Kunt L, Tichy A, Nagyova V, Kybic J. Automatic caries detection in bitewing radiographs: part I - deep learning. Clin Oral Investig. 2023;27(12):7463-71. | Bone loss not the target condition |
| 37 | Tichy A, Kunt L, Nagyova V, Kybic J. Automatic caries detection in bitewing radiographs - part II: experimental comparison. Clin Oral Investig. 2024;28(2):133. | Bone loss not the target condition |
| 38 | Ayhan B, Ayan E, Bayraktar Y. A novel deep learning-based perspective for tooth numbering and caries detection. Clin Oral Investig. 2024;28(3):178. | Bone loss not the target condition |
| 39 | Mao YC, Chen CA, Lin YJ, et al. Automated assessment of peri-implant disease severity by deep learning and image processing in periapical radiographs. J Dent Sci. 2025. Target is peri-implant marginal bone loss, not alveolar bone loss at natural teeth. | Bone loss not the target condition |
| 40 | Lai J, Guo S, Wang K, Li X, Yu X, Wang X. Artificial intelligence for binary dental caries diagnosis using intraoral images and dental radiographs: a systematic review and meta-analysis. BMC Oral Health. 2026;26:437. | Bone loss not the target condition |
| 41 | Atieh MA, Shah M, Hakam A, AlAli F, AlSayed S, Tawse-Smith A, Alsabeeha NHM. Diagnostic accuracy of deep learning models in detecting peri-implant marginal bone loss: a systematic review and meta-analysis. Clin Oral | Bone loss not the target condition |

| No. | Full citation | Principal reason for exclusion |
|---|---|---|
| | Implants Res. 2026;37:385-401. Peri-implant target; the target criterion ranks above publication type. | |
| 42 | Mugri MH. Accuracy of artificial intelligence models in detecting peri-implant bone loss: a systematic review. Diagnostics (Basel). 2025;15(6):655. Peri-implant target; the target criterion ranks above publication type. | Bone loss not the target condition |
| 43 | Li Y, Wang X, Zhu H, Ye W. The diagnostic performance of AI based on dental radiographs in predicting marginal bone loss around dental implants: a systematic review and meta-analysis. J Prosthet Dent. 2025;134(6):2190.e1-2190.e11. Peri-implant target; the target criterion ranks above publication type. | Bone loss not the target condition |
| 44 | Mishra S, Kumar M, Mishra L, Panda S, Panda S, Lewkowicz N, Lapinska B. Estimation of cancellous changes using fractal analysis in patients with periodontitis. Biomedicines. 2023;11(9):2547. | No deep learning component |
| 45 | Ertas K, Pence I, Cesmeli MS, Ay ZY. Determination of the stage and grade of periodontitis according to the current classification of periodontal and peri-implant diseases and conditions (2018) using machine learning algorithms. J Periodontal Implant Sci. 2023;53(1):38-53. Classical machine learning applied to clinician-measured parameters rather than learned image representations. | No deep learning component |
| 46 | Sameer S, Karthik J, Teja MS, Vardhan PA. Estimation of periodontal bone loss using SVM and random forest. In: 2nd International Conference for Innovation in Technology (INOCON). IEEE; 2023. | No deep learning component |
| 47 | Tayman MA, Saricam E. Fractal analysis of trabecular alveolar bone with intrabony and furcation defects using periapical dental radiographs. Eur J Ther. 2024;30(4):456-63. Periapical radiographs and bone as target; the method criterion applies before the in vitro criterion. | No deep learning component |
| 48 | Yadadi SS, Shetty RM, Sowmya V, Bakie W, Jaziri K, Alsaraierh H, et al. Machine learning assisted classification of periodontal health and periodontitis using alveolar bone loss measurements on bitewing radiographs. Front Oral Health. 2026;7:1814025. Random forest applied to clinician-measured bone-loss values rather than learned image representations. | No deep learning component |
| 49 | Alzubaidi L, Zhang J, Humaidi AJ, Al-Dujaili A, Duan Y, Al-Shamma O, et al. Review of deep learning: concepts, CNN architectures, challenges, applications, future directions. J Big Data. 2021;8(1):53. Methodological review with no diagnostic target and no radiographic data. | Review, editorial or conference abstract |
| 50 | Li X, Zhao D, Xie J, Wen H, Liu C, Li Y, Li W, Wang S. Deep learning for classifying the stages of periodontitis on dental images: a systematic review and meta-analysis. BMC Oral Health. 2023;23:1017. | Review, editorial or conference abstract |
| 51 | Ferrara E, Rapone B, D'Albenzio A. Applications of deep learning in periodontal disease diagnosis and management: a systematic review and critical appraisal. J Med Artif Intell. 2025;8:23. | Review, editorial or conference abstract |
| 52 | Khubrani YH, Thomas D, Slator PJ, White RD, Farnell DJJ. Detection of periodontal bone loss and periodontitis from 2D dental radiographs via machine learning and deep learning: systematic review employing APPRAISE-AI and meta-analysis. Dentomaxillofac Radiol. 2025;54:89-108. | Review, editorial or conference abstract |
| 53 | Mahizha SI, Annrose J, Mano Christaine Angelo J, Domilin Shyni I, Veda Giri GV. Deep convolutional neural networks for early detection of interproximal caries using bitewing radiographs: a systematic review. Evid Based Dent. 2025;26:117. | Review, editorial or conference abstract |
| 54 | Lee JE, Choi E, Park JB. Radiographic diagnosis of periodontitis using artificial intelligence: a meta-analysis comparing binary and staging classifications across imaging modalities. BMC Oral Health. 2025;25(1):1921. | Review, editorial or conference abstract |

| No. | Full citation | Principal reason for exclusion |
|---|---|---|
| 55 | Putra RH, Astuti ER, Nurrachman AS, Savitri Y, Vadya AV, Khairunisa ST, Iikubo M. Applicability and performance of convolutional neural networks for the identification of periodontal bone loss in periapical radiographs: a scoping review. Oral Radiol. 2026;42(1):1-12. | Review, editorial or conference abstract |
| 56 | Chiang HM, Jonzen K, Wu WY, Ohberg F, Garoff M, Lovgren A, Lundberg P. How accurate is AI in detecting marginal jaw bone loss? A systematic review and meta-analysis. J Dent. 2025;163:106151. | Review, editorial or conference abstract |
| 57 | Atieh MA, Shah M, Hakam A, Al-Karadsheh O, Zabadi S, AlAli F, Sachdev N, Tawse-Smith A, Alsabeeha NHM. Diagnostic accuracy of artificial intelligence-based deep learning models in detecting furcation involvement: a systematic review and meta-analysis. J Periodontol. 2026;97(7):1559-74. | Review, editorial or conference abstract |
