# Supplementary material for: Deep Learning for the Assessment of Alveolar Bone Loss on Intraoral Radiographs: A Systematic Review
Source: Diagnostics (Basel). 2026 Aug 14;16(16):2575. doi: 10.3390/diagnostics16162575 (PMC13512858; doi:10.3390/diagnostics16162575)
Supplement: Supplementary file 1 [file diagnostics-16-02575-s001.zip › S6-chronology-deviations.pdf]

Supplementary File S6

Chronology of the review and deviations from the registered protocol

Deep Learning for the Assessment of Alveolar Bone Loss on Intraoral Radiographs: A Systematic Review. Manuscript diagnostics-4481953.

Table S6.1. Chronology.

Stage	Dates	Detail
Protocol drafting and pilot scoping	2-8 June 2026	Eligibility criteria drafted; pilot screening of 50 records; criteria clarified and reviewers recalibrated.
Electronic searches	9-12 June 2026	Seven bibliographic databases plus citation chasing, preprint servers, grey literature and hand-searching. Final search date 12 June 2026.
Title and abstract screening	13-24 June 2026	946 records screened in duplicate in Rayyan with blinding enabled; Cohen kappa 0.86.
Full-text assessment and data extraction	25 June - 5 July 2026	73 full texts assessed in duplicate; Cohen kappa 0.91; extraction into the master study list.
Risk of bias assessment	6-17 July 2026	QUADAS-2 with AI-specific signalling questions, completed independently in duplicate.
PROSPERO registration	20 July 2026	Registered as CRD420261455818. Registration was retrospective.
APPRAISE-AI appraisal (post hoc addition)	24-26 July 2026	Added after registration; recorded as a protocol deviation below.
PROSPERO amendment (version 1.1)	30 July 2026	Stage-of-review declaration and review start date corrected. Both versions remain publicly visible in the PROSPERO version history.
First revision	July 2026	Response to reviewer reports; all quality appraisals completed; master study list constructed.
Second revision: verification against source articles	July-August 2026	Every study-level value re-read from the source publications; Table 4 completed; two included reports reclassified as ineligible; excluded-study list re-audited in full.

Table S6.2. Deviations from the registered protocol.

Deviation	Description
Registration timing	The protocol was registered on 20 July 2026, after searching, screening, extraction and risk-of-bias assessment had been completed. Registration was therefore retrospective. No separate protocol document exists; the PROSPERO record is the protocol of record.
Meta-analysis not performed	The registered protocol allowed for a bivariate hierarchical meta-analysis if a sufficiently homogeneous subset emerged. No such subset emerged, and findings are synthesised narratively following SWiM guidance. The reasons are set out per outcome in Table 9.
APPRAISE-AI added post hoc	APPRAISE-AI was not named in the registered protocol. It was added because QUADAS-2 and CLAIM together do not capture the reproducibility and data-quality domains specific to artificial intelligence studies.
Reclassification of two reports at revision	Two reports initially retained were re-examined against their full texts and found ineligible: one used panoramic radiographs exclusively (modality criterion), and one evaluates tooth-number recognition and full-mouth-series arrangement rather than alveolar bone loss (target-condition criterion). Both appear in Supplementary File S5.
Re-audit of the excluded-study list	Every excluded full-text report was re-verified against its full text and reassigned under the predefined hierarchy. The audit recovered two reports assessed at full text but omitted from the list, giving 73 reports assessed for eligibility rather than 71, and reassigned records previously placed in the "no extractable performance metric" and "duplicate report" categories, both of which are now empty.

PROSPERO record CRD420261455818, version 1.1, published 30 July 2026.
