# Supplementary material for: Deep Learning for the Assessment of Alveolar Bone Loss on Intraoral Radiographs: A Systematic Review
Source: Diagnostics (Basel). 2026 Aug 14;16(16):2575. doi: 10.3390/diagnostics16162575 (PMC13512858; doi:10.3390/diagnostics16162575)
Supplement: Supplementary file 1 [file diagnostics-16-02575-s001.zip › S7-reference-standards.pdf]

Supplementary File S7

Classification of reference standards for all included publications

Deep Learning for the Assessment of Alveolar Bone Loss on Intraoral Radiographs: A Systematic Review. Manuscript diagnostics-4481953.

For every included publication the reference standard was recorded as described in the source article, together with the QUADAS-2 judgements for the reference-standard domain. No included publication used a three-dimensional, surgical or histological reference standard: in every case the ground truth was expert interpretation of the same two-dimensional radiograph on which the model was evaluated.

Table S7.1. Reference standard for each of the 16 included publications.

Study	Reference standard as reported	QUADAS-2 risk of bias: reference standard	QUADAS-2 applicability: reference standard
Lee JH 2018	Clinical diagnosis by periodontists integrated with radiographic assessment.	Low	Low
Moran 2021	Expert-labelled periodontal bone-loss regions.	Unclear	Low
Danks 2021	Manual CEJ, bone-level and apex landmarks by calibrated clinicians.	Low	Low
Chen H 2021	One dentist with >5 years' experience; no calibration, second reader or adjudication.	High	High
Khan 2021	Three specialists; one annotated and two reviewed; cases excluded when at least two did not agree.	Low	Low
Alotaibi 2022	Two examiners with consensus grading.	Low	Low
Lee C-T 2022	Independent calibrated examiners and 2018 periodontal classification.	Low	Low
Tsoromokos 2022	Manual measurements by calibrated examiners; reliability reported.	Low	Low
Chen C-C 2023	Expert annotations; calibration detail limited.	Unclear	Low
Dujic 2023	Trained/calibrated dentists using four-category PBL grading.	Low	Low
Hoss 2023	Trained/calibrated dentists using the same four-category PBL grading as Dujic.	Low	Low
Chen I-H 2024	Periodontist-assigned staging; ICC reported.	Low	Low
Yavuz 2024	Calibrated examiner annotation with high kappa values.	Low	Low
Erturk 2025	Radiologist classification using 2018 staging thresholds.	Low	Low
AlGhaihab 2025	Two calibrated examiners using AAP/EFP classification; diagnostic accuracy with 95% CIs.	Low	Low
Bayirli 2026	Multiple independent expert clinicians and consensus ground truth.	Low	Low

Agreement statistics. Inter- or intra-examiner agreement was reported as a statistic by a minority of publications. On the largest dataset in the field, inter-examiner Cohen kappa for the four-category bone-loss reference standard was reported as 0.454 to 0.482, with intra-examiner agreement 0.739; on the conventional interpretation these represent moderate rather than substantial agreement, and they bound what any model trained on that standard can be shown to achieve.

Blinding. No included publication reported whether annotators were blinded to clinical information or to model output. This is recorded as a signalling-question response in Supplementary File S3.
